# The *Caulobacter crescentus* DciA promotes chromosome replication through topological loading of the DnaB replicative helicase at replication forks

**DOI:** 10.1093/nar/gkac1146

**Published:** 2022-12-09

**Authors:** Shogo Ozaki, Dengyu Wang, Yasutaka Wakasugi, Naoto Itani, Tsutomu Katayama

**Affiliations:** Department of Molecular Biology, Graduate School of Pharmaceutical Sciences, Kyushu University, Higashi-ku, Fukuoka 812-8582, Japan; Department of Molecular Biology, Graduate School of Pharmaceutical Sciences, Kyushu University, Higashi-ku, Fukuoka 812-8582, Japan; Department of Molecular Biology, Graduate School of Pharmaceutical Sciences, Kyushu University, Higashi-ku, Fukuoka 812-8582, Japan; Department of Molecular Biology, Graduate School of Pharmaceutical Sciences, Kyushu University, Higashi-ku, Fukuoka 812-8582, Japan; Department of Molecular Biology, Graduate School of Pharmaceutical Sciences, Kyushu University, Higashi-ku, Fukuoka 812-8582, Japan

## Abstract

The replicative DNA helicase translocates on single-stranded DNA to drive replication forks during chromosome replication. In most bacteria the ubiquitous replicative helicase, DnaB, co-evolved with the accessory subunit DciA, but how they function remains incompletely understood. Here, using the model bacterium *Caulobacter crescentus*, we demonstrate that DciA plays a prominent role in DNA replication fork maintenance. Cell cycle analyses using a synchronized *Caulobacter* cell population showed that cells devoid of DciA exhibit a severe delay in fork progression. Biochemical characterization revealed that the DnaB helicase in its default state forms a hexamer that inhibits self-loading onto single-stranded DNA. We found that upon binding to DciA, the DnaB hexamer undergoes conformational changes required for encircling single-stranded DNA, thereby establishing the replication fork. Further investigation of the functional structure of DciA revealed that the C-terminus of DciA includes conserved leucine residues responsible for DnaB binding and is essential for DciA *in vivo* functions. We propose that DciA stimulates loading of DnaB onto single strands through topological isomerization of the DnaB structure, thereby ensuring fork progression. Given that the DnaB-DciA modules are widespread among eubacterial species, our findings suggest that a common mechanism underlies chromosome replication.

## INTRODUCTION

**Figure 1.**
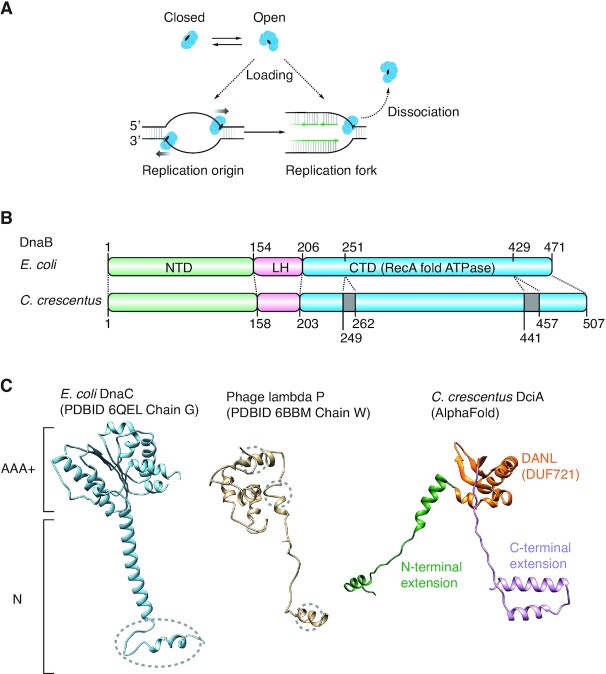
A model for replicative DNA helicase loading. (**A**) A model for replicative helicase loading in *E. coli*. The replicative DnaB_EC_ helicase (blue circles) forms a hexameric, closed ring as a default state. At the replication origin or stalled replication forks, the DnaB_EC_ hexamer undergoes conformational changes to open the ring structure, thereby allowing it to engage ssDNA. (**B**) Comparison of DnaB_EC_ and *C. crescentus* DnaB. The two sequences were aligned using ClustalW and are shown schematically. The overall similarity at the amino acid level was 52%. (**C**) Structural comparison among *E. coli* DnaC (PDBID 6QEL, Chain G), phage lambda P (PDBID 6BBM, Chain W), and a predicted *C. crescentus* DciA structure generated by AlphaFold. The central DANL domain (amino acids 47–123; DUF721 in Pfam), the N-terminal extension (amino acids 1–46), and the C-terminal extension (amino acids 124–179) are highlighted in different colors. Previously determined *E. coli* DnaB binding regions are indicated by dotted circles. The different domains of DciA are highlighted in different colors.

The unwinding of double-stranded (ds) DNA is fundamental for the semiconservative replication of chromosomes in all cellular life (1–3). In the bacterial kingdom, ubiquitous replicative DnaB helicases are installed at a single, unique origin of replication during initiation of replication (4,5). The bacterial replication origin encodes instructions for the regulated assembly of the DnaA initiator proteins to form an initial complex (6–11). This complex locally unwinds the dsDNA and loads the DnaB helicases onto the resulting single-stranded (ss) ‘replication bubble’ (Figure 1A). Subsequent bidirectional translocation of the loaded DnaB helicases expands the ssDNA region, allowing for recruitment of replisome components and formation of the replication forks, thereby initiating chromosome replication (12). Along with DNA unwinding, DnaB loading plays a crucial role in the robust progression of replication forks (13,14). The DnaB helicase can accidentally dissociate from the fork, especially when it encounters lesions or other obstacles on the DNA (Figure 1A). Rescuing the collapsed fork necessitates re-installation of the DnaB helicases. Thus, temporal and spatial regulation of replicative helicase loading plays an essential role in the completion of chromosomal replication. However, the dedicated mechanisms by which this process is regulated, both at the replication origin and at the forks are still unclear.

One of the best characterized helicase loading systems in bacteria comprises *Escherichia coli* DnaB (DnaB_EC_) and its loading partner, DnaC (1–3). DnaB_EC_ forms a ring-shaped homohexamer, encircles ssDNA, and drives DNA unwinding in the 5′ to 3′ direction using the energy of ATP hydrolysis (15–18). The DnaB_EC_ protein comprises three functional domains: the N-terminal domain, the central linker domain, and the C-terminal RecA-fold ATPase domain (Figure 1B; Supplementary Figure S1) (1,2,18–20). Of these, the linker and C-terminal domains of DnaB_EC_ include specific sites for interaction with the DnaC loader (17,21,22). DnaC, an AAA + ATPase family protein, is essential for both the initiation and elongation processes of chromosomal replication in *E. coli* (Figure 1C) (23,24). The DnaC monomers bind to a DnaB_EC_ hexamer to form a 1:1 DnaB_EC_-DnaC complex (17,21,25). While the DnaB_EC_ hexameric ring is essentially closed in the absence of DnaC, the DnaB_EC_-DnaC complex undergoes a conformational change to form an open-ring structure competent to engage ssDNA (17,21,25). Subsequent dissociation of DnaC from the complex allows translocation of the ssDNA-loaded DnaB_EC_ hexamer along the DNA toward the replication terminus (23,26). Recent cryogenic electron microscopy and crystal structure analyses demonstrate that the DnaC N-terminus specifically interacts with the exterior surface of the DnaB hexameric ring, which then imposes accumulated distortions on the closed ring to allow it to switch into the open conformation (17,21). A similar mechanism is proposed for the phage lambda P protein, which has little or no homology to DnaC (Figure 1C) (27). Importantly, while the counterpart of DnaC is conserved as DnaI in *Bacillus subtilis* (28), the vast majority of bacterial genomes do not encode either DnaC/DnaI or P homologs (29). Thus, how far the molecular mechanisms underlying the DnaC-DnaB system are conserved in bacterial evolution, remains unclear.

Recent bioinformatic analyses have exposed the DciA family protein as a potent operator of the replicative helicase in bacterial species that lack a DnaC/DnaI homolog (29–31). Despite substantial diversity in protein sequences, this family is defined by the presence of the DANL domain, which displays structural homology to the N-terminal domain of DnaA, the C-terminal domain of the DNA polymerase tau subunit, and the flagellar hook-length controlling FliK protein (Figure 1C). Although the crystal structure of the DciA family proteins has not been solved, the structural homology of the DANL domain has been supported by a recent analysis on nuclear magnetic resonance chemical shift data for the backbone of the truncated DANL domain from the *Vibrio cholerae* DciA homolog (31). Characterization of DciA homologs from *Pseudomonas aeruginosa*, *Mycobacterium tuberculosis*, and *Vibrio cholerae* suggested that the DciA family protein directly binds the cognate DnaB homolog to modulate helicase activities through an unknown molecular mechanism. A recent small angle X-ray scattering analysis for the *V. cholerae* system suggested that the cognate DciA and DnaB proteins interact in 1:2 stoichiometry. Yet, functional domains responsible for this interaction have been unexplored (31). Moreover, in *P. aeruginosa* and *M. tuberculosis*, inactivation of DciA family proteins results in production of many abortive cells compromised for DNA replication, highlighting their essential role in the process of DNA replication (29,30). However, these studies were carried out using asynchronous cell populations; thus, the direct and indirect consequences of the absence of the DciA family proteins could not be clearly defined. Therefore, spatiotemporal analysis of DciA-family proteins to address their essential role in DNA replication and determine the underlying molecular mechanisms is vital for understanding the helicase loading system in bacterial species that encode the DciA family.

Here, we provide evidence that the DciA-DnaB system plays a primary role in DNA elongation in the model organism, *Caulobacter crescentus*, an aquatic Gram-negative alphaproteobacterium lacking a DnaC-family protein. In this bacterium, cell cycle progression generates two genetically identical but physiologically distinct progeny cells. The stalked progeny is sessile and can initiate chromosome replication (entry into S phase), whereas the motile swarmer progeny blocks replication initiation and consequently experiences an extended nonproliferating period, termed the G1 phase (11,32–36). By analyzing synchronized G1 phase cells, we revealed that while *C. crescentus* cells devoid of DciA are able to initiate DNA replication, DNA elongation is perturbed, resulting in cell death. Mechanistically, we demonstrate that physical interaction of *C. crescentus* DciA and DnaB imposes the conformational changes on the cognate DnaB necessary to achieve its loading onto ssDNA. Furthermore, we uncover that the conserved leucine residues at the DciA C-terminus are crucial for DnaB binding. These data demonstrate that the *C. crescentus* DciA-DnaB system mechanistically resembles the *E. coli* DnaB-DnaC system, despite structural dissimilarity between DciA and DnaC. Given the strict requirement of *C. crescentus* DciA for DNA elongation, we propose that DciA-mediated helicase loading plays a central role in ensuring replication fork progression.

## MATERIALS AND METHODS

### Bacterial strains and DNAs

The strains, plasmids, and primers used in this study are listed in Tables 1, 2 and 3, respectively. *Caulobacter* strains were grown at 30°C in PYE (2 g/l peptone, 1 g/l yeast extract, 0.8 mM magnesium sulfate and 0.5 mM calcium chloride) medium supplemented with appropriate antibiotics, as described. When necessary, cumate (1 μM), glucose (0.1%), or xylose (0.1%) was added to the culture medium, as indicated. For synchronization experiments, newborn G1 cells were harvested using the LUDOX density-gradient centrifugation method (37,38). The isolated cells were washed twice in ice-cold PYE, and released into prewarmed PYE at 30°C.

**Table 1.** Strains used in this study

Strain	Genotype	Reference
*Caulobacter crescentus*		
NA1000	wild-type *Caulobacter crescentus* strain	(61)
SHQ209	NA1000 *dciA*::P*xylX*-*dciA*	This study
SHQ254	NA1000 *xylX*::*sspB_ec_*	This study
SHQ258	SHQ254 *dciA*::*pMCS4dciAssrA*	This study
SHQ259	SHQ254 *dnaA*::*pMCS4dnaAssrA*	This study
		
*Escherichia coli*		
DH5a	General cloning strain	Invitrogen
Rosetta™ 2(DE3)	Strain for overexpression of recombinant protein	Novagen

**Table 2.** Plasmids used in this study

Plasmid	Description	Reference
pBXMCS2sfTq2	A pBXMCS2 derivative expressing sfTq2	(39)
pET21a	An ampicillin-resistant pET expression vector	Novagen
pET21aHisdciA	A ET21a derivative with HisDciA	This study
pET21aHisdciA(L167S)	A pET21aHisdciA derivative with the *L167S* allele	This study
pET28-sfTq2dciA(WT)-dnaBcHis	A pET28a derivative co-expressing sfTq2-DciA and DnaB-His	This study
pET28a	A kanamycin-resistant pET expression vector	Novagen
pET28a-hisdnaAcc	A pET28a derivative with HisDnaA	This study
pET28aCCNA01737cHis	A pET28a derivative expressing CCNA_01737 with a C-terminal His6 tag	This study
pET28aDnaBcHis(K234A)	A pET28aCCNA01737cHis derivative expressing DnaB(K234A)	This study
pETsfTq2dciA(124–179)-d	A pET28a derivative expressing sfTq2-DciA(124–179)	This study
pETsfTq2dciA(2–47)-d	A pET28a derivative expressing sfTq2-DciA(2–47)	This study
pETsfTq2dciA(46-127)-d	A pET28a derivative expressing sfTq2-DciA(46-127)	This study
pETsfTq2dciA(WT)-d	A pET28a derivative expressing sfTq2-DciA	This study
pETsfTq2dciA(L163S)-d	A pETsfTq2dciA(WT)-d derivative with the *dciA*(L163S) allele	This study
pETsfTq2dciA(L167S)-d	A pETsfTq2dciA(WT)-d derivative with the *dciA*(L167S) allele	This study
pETsfTq2dciA(L170S)-d	A pETsfTq2dciA(WT)-d derivative with the *dciA*(L170S) allele	This study
pLacQFlacImCherry	A pNPTS138 derivative to introduce the *lacImCherry* ORF into the chromsomal lacA locus	(40)
pMCS-4	An integrating plasmid with gentamycin-resistance	(62)
pMCS4dciAssrA	An integrating plasmid with *dciA-ssrA*	This study
		This study
pMCS4dnaAssrA	An integrating plasmid with *dnaA-ssrA*	This study
pMR10	A kanamycin-resistant RK2 plasmid	(63)
pMR10dciA	A pMR10 derivative expressing wild-type DciA	This study
pMR10dciA(L163S)	A pMR10dciA derivative with the *dciA(L163S)* allele	This study
pMR10dciA(K164A)	A pMR10dciA derivative with the *dciA(K164A)* allele	This study
pMR10dciA(Q165A)	A pMR10dciA derivative with the *dciA(Q165A)* allele	This study
pMR10dciA(L167S)	A pMR10dciA derivative with the *dciA(L167S)* allele	This study
pMR10dciA(L168S)	A pMR10dciA derivative with the *dciA(L168S)* allele	This study
pMR10dciA(K169A)	A pMR10dciA derivative with the *dciA(K169A)* allele	This study
pMR10dciA(L170S)	A pMR10dciA derivative with the *dciA(L170S)* allele	This study
pMR10dciA(R171A)	A pMR10dciA derivative with the *dciA(R171A)* allele	This study
pMR10dciA(V173A)	A pMR10dciA derivative with the *dciA(V173A)* allele	This study
pMR10dciA(L174A)	A pMR10dciA derivative with the *dciA(L174A)* allele	This study
pMR10dciA(S175A)	A pMR10dciA derivative with the *dciA(S175A)* allele	This study
pMR10dciA(E177A)	A pMR10dciA derivative with the *dciA(E177A)* allele	This study
pMR10dciA(R178A)	A pMR10dciA derivative with the *dciA(R178A)* allele	This study
pMR10HAdciA	A pMR10 derivative expressing N-terminal HA-tagged DciA	This study
pMR10HAdciA160	A pMR10HAdciA derivative with the ΔC9 allele	This study
pMR10HAdciA170	A pMR10HAdciA derivative with the ΔC19 allele	This study
pMR20	A tetracycline-resistant RK2 plasmid	(64)
pMR20dciA	A pMR20 derivative with dciA	This study
pNPTS-Pxyl-sspB	A pNPTS138 derivative to introduce sspBec at the xylX locus	This study
pNPTS-PxyldciA	A pNPTS138 derivative to introduce the xylose-dependent promoter at the dciA locus	This study
pNPTS138	A kanamycin-resistant suicide vector	(37)
pNPTSPxylDivKpleD	a pNPTS derivative containing the PxylX promoter	(37)
pNPTSxylXlacImCherry	a pNPTS138 derivative to introduce the lacImCherry ORF into the chromsomal xylX locus	This study
pQF	A low-copy-number tetracycline-resistance-conferring vector with the cumate-dependent promoter	(65)
pQFdciA	A pQF derivative with *dciA*	This study

**Table 3.** Oligonucleotides used in this study

Name	Sequence (5′-)
102	TTCGGTACCCTGACCGCGCTGGTCGGGGAGG
103	GTTCTCGAGGCCCCGCAGCTTGCGCGTCAGGGTC
191	CTAAAGCTTTCGCATGCGTCGCCCCCTGCCCACG
192	TCCGAGCTCTAGCGCTCCGAAGACAGGACCC
196	GAAAAGCTTCGATCCTGCGCAGCAAGCGGACTAA
197	CCGCTCGAGGCGCTCCGAAGACAGGACCCCGCGA
291	GCCAAGCTTGTCAATGGTCCGATCCCGTCTC
292	GCATGTACCCATACGATGTTCCAGATTACGCTGGAGGGGGGGGATCCCGTCGCCCCCTGCCCACGCCGGAAG
293	CGACGGGATCCCCCCCCTCCAGCGTAATCTGGAACATCGTATGGGTACATGCGAGGAAACTAGCATGGGGCC
294	ACCGAATTCGGTGCCCGGAAAGACGCCGCCG
307	GGGACTAGTCGCCGGGGCGATCGTCGGTGAC
311	CGGCGGCATGGACGAGCTGTACAAGGGAGGCGGTGGATCCCGTCGCCCCCTGCCCACGCCGGAAG
312	ACCGAATTCATGAGCGTGAGCAGGCCCGCCTT
362	TTTGGATCCCTTGTCATCGTCATCCTTGTAATCGATATCATGATCTTTATAATCACCGTCATGGTCTTTGTAGTCCATGCGAGGAAACTAGCATGGGGCC
392	CGTGGTACCAGGCCAGCAGGGCCGAGCGG
393	GCCAAGCTTGTCAATGGTCCGATCCCGTCTC
425	TTCCCATGGGGAGTAAAGGAGAAGAACTTTTCACTG
426	CATGGATCCGCCAGAACCAGCAGCGGAGCCAG
467	GCACTAGTTCGGCGCGCCGCCCGCTTCC
468	CATCGTATAACGTTACTGGTTTCATATGGTCGTCTCCCCAAAACTCGAGCGTC
471	CATGGACGAGCTGTACAAGTAGGAATTCCTGCCGGAAGATTGGAAAG
472	TGGGCATGCTGAGCAGCAGGGCCGAACGACC
575	GTAGGTACCGCGAGGAAACTAGCATGGGGCCGCC
576	AAGCATATGCGTCGCCCCCTGCCCACGCCGGAAG
577	CGAGGTACCCTCTAGAACTAGTGGATCCAAATAA
578	TGCAGGCTGCCTTTCCAGCAGGCGC
606	ATCAAGCTTCTAGCGCTCCGAAGACAGGACCCCG
607	TAACATATGCCGATGTCTCTCGTCCCTGCGCT
608	GCCGCGGCCGCTCGTCCGACGGAATATTCCGCGCC
708	CAAGGATCCAGCCGGGCAGTGCCCGGCTTTTCTC
709	TGCGGATCCTTATTTTTGCTGCTGCGCGTTCCAG
738	GGGGGGGGATCCCGTCGCCCCCTGCCCA
739	CCCAAGCTTACAGCTTCAGCAGGGCCTGTTTGAGC
740	TGAAAGCTTAGTCGGGCTGTTCGGCCAGGCTGTCG
770	GATGGGGAATTGTTATCCGCTCACAATTCCCCTATAGTGAGTCGTATTAATTTCGCTAGCGCTCCGAAGACAGGACCCCG
780	ATCCATATGGATTTGTCACAGCTAACACCA
781	CCTGAATTCTATTACTTCACAACGCGTAATGCCG
782	TCGAGGCCGCAAACGACGAAAACTACTCCGAAAACTACGCTGACGCAAGCTG
783	AATTCAGCTTGCGTCAGCGTAGTTTTCGGAGTAGTTTTCGTCGTTTGCGGCC
784	CGCCGCAAACGACGAAAACTACTCCGAAAACTACGCTGACGCAAGCTG
785	AATTCAGCTTGCGTCAGCGTAGTTTTCGGAGTAGTTTTCGTCGTTTGCGGCGAGCT
808	CCCTCTAGAAATAATTTTGTTTAACTTTAAGAAGGAGATATACCATGGGCAGCAGCCATCACCATCATCATCACCGTCGCCCCCTGCCCACGCCGGAAG
864	TTTTTTTTTTTTTTTTTTTTTTTTTTTTTTTGCCCTGTGGATAACAAGGATCCGGCTTTTAAGATCAACAACCTGGAAA
871	TTTCCAGGTTGTTGATCTTAAAAGCCGGATCCTTGTTATCCACAGGGCTTTTTTTTTTTTTTTTTTTTTTTTTTTTTTT
872	TTTCCAGGTTGTTGATCTTAAAAGCCGGATCCTTGTTATCCACAGGGC
894	TCGGAGAACTATATCGCACAATTTTTTTTTTTTTTTTTTTTTTTTTTTTTTTTTTTTTTCTGCCCTGTGGATAACAAGGA
895	TCCTTGTTATCCACAGGGCAGTTTTTTTTTTTTTTTTTTTTTTTTTTTTTTTTTTTTTTTTGTGCGATATAGTTCTCCGA
896	TCGGAGAACTATATCGCACAA
897	CTGCCCTGTGGATAACAAGGA
1100	GGCGATGTTGGTCGCCAGGGCCGTCGCACCCATCGAGGGGCGTCCGGCCAGG
1101	CCTGGCCGGACGCCCCTCGATGGGTGCGACGGCCCTGGCGACCAACATCGCC
1169	GTAGGATCCCGTCGCCCCCTGCCCACGCCGGAA
1170	GCGGCTAGCTACTTGCCGAAGCGGTCTTCCAGGTCC
1171	GACGGATCCGGCAAGGGTCCCGCCGCGCTGCAG
1172	GGCGCTAGCCTACTTGACCGGGCCCTGCACGATCCGC
1173	ATCGGATCCGGCCCGGTCAAGGCGCCCGCCGCCGCG
1393	GGCCGCAAGCTTCTAGCGCTCCGAAGACAGGACCCCGGCACCCAGCTTCAGCAGGGCC
1394	GGCCGCAAGCTTCTAGCGCTCCGAAGACAGGGCCCCGCGACCCAGCTTCAGC
1395	GGCCGCAAGCTTCTAGCGCTCCGAAGACGCGACCCCGCGACCCAGCTTCA
1396	GGCCGCAAGCTTCTAGCGCTCCGAAGCCAGGACCCCGCGACCCAGCT
1397	GGCCGCAAGCTTCTAGCGCTCCGCAGACAGGACCCCGCGACCCA
1398	GGCCGCAAGCTTCTAGCGCGCCGAAGACAGGACCCCGCGA
1399	GGCCGCAAGCTTCTAGGCCTCCGAAGACAGGACCCCGC
1400	TCGGAGAACTATATCGCACAATTTTTTTTTTTTTTTTTTTTTTTTTTTTCTGCCCTGTGGATAACAAGGA
1401	TCCTTGTTATCCACAGGGCAGTTTTTTTTTTTTTTTTTTTTTTTTTTTTTTGTGCGATATAGTTCTCCGA
1402	TCGGAGAACTATATCGCACAATTTTTTTTTTTTTTTTTTCTGCCCTGTGGATAACAAGGA
1403	TCCTTGTTATCCACAGGGCAGTTTTTTTTTTTTTTTTTTTTGTGCGATATAGTTCTCCGA
1416	GGTCGCGGGGTCCTGTCTTCGGAGC
1417	CAGCTTCAGCAGGGCCTGTTTGGACGCGCCCGTCGGGCTGTTCGGCCAG
1418	CAGCTTCAGCAGGGCCTGTGCGAGCGCGCCCGTCGGGCTGTTCGGC
1419	CAGCTTCAGCAGGGCCGCTTTGAGCGCGCCCGTCGGGCTGTTC
1420	CAGCTTCAGCGAGGCCTGTTTGAGCGCGCCCGTCGGG
1421	CAGCTTCGACAGGGCCTGTTTGAGCGCGCCCGTC
1422	CAGCGCCAGCAGGGCCTGTTTGAGCGCGCCC
1423	CGACTTCAGCAGGGCCTGTTTGAGCGCG

Detailed procedures for construction of the strains and plasmids are described in the Supplemental Materials.

### Flow cytometry

This assay was performed essentially as described previously (39,40). In brief, exponentially growing cells (100 μl) were fixed in ice-cold 70% ethanol, washed, and resuspended in buffer supplemented with 2 μM SYTOX Green (Thermo Fisher Scientific). The fluorescent intensity and light scattering were analyzed using the FACS Calibur system (BD Biosciences).

### Western blotting

The polyclonal anti-DciA antibody generated using the recombinant DciA protein was purchased form SCRUM co. The anti-DciA (1:1000) antibody was used as primary antibody, which was detected by AP-conjugated anti-rabbit second antibodies (Bio-rad). The immune reactions were performed in Immunoreaction Enhancer Solution (Toyobo) according to the manufacturer's instructions.

### Recombinant proteins

Recombinant *C. crescentus* proteins were expressed in *E. coli* and purified as described in the Supplemental Material. *E. coli* proteins (DnaB_EC_ and DnaC) were purified as described previously (41).

### Size exclusion chromatography

This assay was performed essentially as described previously (22,39). Briefly, proteins were loaded onto a Superdex 200 Increase 10/300 GL column (24 ml column volume), equilibrated with SEC buffer (25 mM Tris–HCl [pH 7.5], 300 mM sodium chloride, 20% glycerol, 0.1 mM ATP and 5 mM magnesium chloride), and fractionated at a flow rate of 0.2 ml/min.

### *In vitro* assays using recombinant proteins

The *in vitro* unwinding assay using a forked DNA substrate was performed essentially as described previously (22). The DNA substrate comprised 48 bp duplex DNA flanking 31-mer single-stranded tails. DnaB-His, His-DciA, and the DNA substrate (12.5 nM) were incubated at 30°C in buffer (20 mM Tris–HCl [pH 7.5], 10 mM magnesium acetate, 50 mM potassium glutamate, 0.1 mg/ml bovine serum albumin, and 2 mM ATP) containing 48-mer synthetic oligonucleotide 872 (62.5 nM) as a competitor. After reactions were terminated by addition of phenol/chloroform/isoamyl alcohol solution, DNA samples were extracted and analyzed using 6% polyacrylamide gel electrophoresis. The substrate was generated by annealing synthetic DNA strands 864 and fluorescently-labeled 871.

For the helicase loading assay using a replication bubble-mimetic DNA substrate comprising a ssDNA bubble intervened by 21 bp duplex DNA regions, the annealed product of synthetic oligonucleotides (894/895 for 38-mer ssDNA bubble; 1400/1401 for 28-mer ssDNA bubble; 1402/1403 for 18-mer ssDNA bubble) was used instead of a forked DNA substrate, and competitor DNA was replaced by a mixture of fluorescently-labeled 21-mer oligonucleotides (896 and 897; 31.3 nM each). DNA samples were extracted and analyzed using 9% polyacrylamide gel electrophoresis.

When radioactively-labelled substrates were used, the synthetic DNA strands (864, 894, 1400 and 1402) were incubated with T4 polynucleotide kinase (Toyobo) and [γ-^32^P]ATP according to the manufacturer's instructions, followed by annealing with the complementary strands.

The ATPase assay was performed essentially as described previously (16). Briefly, DnaB-His and HisDciA were incubated in buffer (20 mM Tris–HCl [pH 7.5], 10 mM magnesium acetate, 50 mM potassium glutamate, 0.1 mg/ml bovine serum albumin) containing various concentrations of ATP for 5 or 10 min at 30°C. Reactions were terminated by addition of a solution (10 mM Tris–HCl [pH 8.0], 1mM EDTA, 0.1% SDS, 1 mM ATP and 1 mM ADP), and a portion (0.5 μl) was spotted on a TLC PEI Cellulose plate (Merck). The plate was developed using a 1 M HCOOH/0.5 M LiCl solution.

To perform *in vitro* pulldown assays, Rosetta™ 2(DE3) cells harboring pETsfTq2dciA(WT)-d or its derivatives were grown exponentially in PY (10 g/l peptone, 5 g/l yeast extract, and 5 g/l sodium chloride) medium (200 ml) supplemented with kanamycin and chloramphenicol at 30°C, and expression of sfTq2-tagged proteins was induced at 30°C for 1 h by addition of 1 mM isopropyl-β-d-thiogalactoside. Cells were harvested by centrifugation and resuspended in Lysis buffer to adjust the cell density to OD_600_ of 200. Following cell lysis using lysozyme (0.2 mg/ml) and brief sonication, cleared cell lysate was prepared using ultracentrifugation. The sfTq2-tagged proteins were further purified through ammonium sulfate (0.25 g/ml) precipitation. Precipiate was dissolved in lysis buffer and a portion containing 4 μg of the sfTq2-tagged proteins and purified DnaB-His (6 μg) were incubated with Ni-conjugated magnetic beads (Promega) for 20 min at 4°C in R1 buffer. After washing the resin twice using W1 buffer, materials retained on the resin were eluted in Elution buffer (10 μl). A portion (2.5 μl) of the eluted sample was analyzed using SDS 12% polyacrylamide gel electrophoresis and Coomassie brilliant blue staining. Buffers used are as follows: Lysis buffer (25 mM Tris–HCl [pH 7.5], 10 mM magnesium acetate, 300 mM sodium chloride, 0.1% Triton X-100, and 5% glycerol), R1 buffer (25 mM Tris–HCl [pH 7.5], 10 mM magnesium acetate, 300 mM sodium chloride, 0.01 mM ATP and 5% glycerol), W1 buffer (25 mM Tris–HCl [pH 7.5], 10 mM magnesium acetate, 400 mM sodium chloride, 0.01 mM ATP, 40 mM imidazole and 5% glycerol), and Elution buffer (Lysis buffer with 500 mM imidazole).

## RESULTS

### DciA is required for *C. crescentus* growth

**Figure 2.**
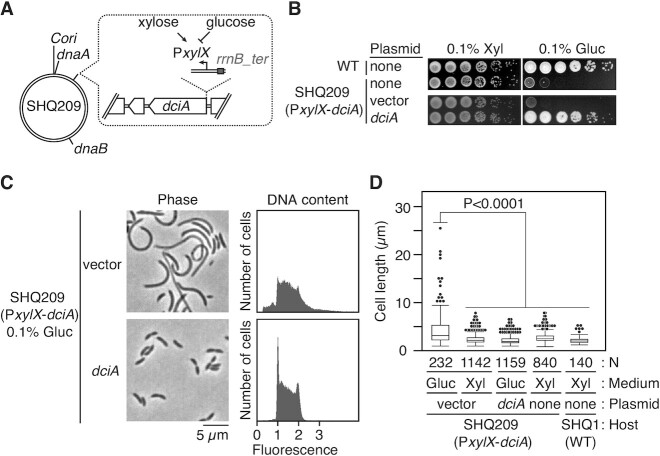
DciA is essential for *C. crescents* proliferation (**A**) Schematic representation of a DciA-depletable strain (SHQ209), in which the xylose-dependent promoter (P*xylX*) flanking the *E. coli rrnB* T1T2 transcriptional terminator (*rrnB_ter*) is integrated at the *dciA* promoter locus. The relative positions of *Cori*, *dnaA*, *dciA*, and *dnaB* are indicated on the circular *C. crescentus* genome. (**B**) Colony formation in the *PxylX-dciA* strain. The wild-type NA1000 (WT) and SH209 strains were grown overnight at 30°C in PYE medium supplemented with 0.1% xylose. Five-fold serial dilutions of the overnight culture were spotted on PYE agar supplemented with 0.1% xylose or 0.1% glucose, followed by incubation at 30°C for 2d. When the SHQ209 strain harboring pMR10 (vector) or pMR10-HAdciA (*dciA*) was analyzed, kanamycin was added to the medium. (**C**) Cell morphology and DNA content of the *PxylX-dciA* strain. The SHQ209 strain harboring pMR10 (vector) or pMR10-HAdciA (*dciA*) was grown for 9 h at 30°C in PYE medium supplemented with 0.1% glucose and kanamycin, followed by fixation in 70% ethanol. After DNA staining with SYTOX Green, cell morphology and DNA content were analyzed using phase-contrast microscopy and flow cytometry, respectively. (**D**) The distributions of cell lengths are shown using a box plot. The p-value was calculated using the Mann–Whitney–Wilcoxon test.

To determine whether *dciA* (namely, CCNA_00380) is an essential gene in *C. crescentus*, we investigated the colony forming ability of a *C. crescentus* strain depleted of DciA. To express DciA conditionally, we engineered the P*xylX*-*dciA* strain in which the xylose-dependent promoter (P*xylX*) was inserted upstream of the *dciA*-coding region (Figure 2A). In the presence of the inducer, xylose, the P*xylX*-*dciA* strain maintained colony forming ability similar to that of the wild-type NA1000 strain (Figure 2B). By contrast, when the medium was supplemented with glucose instead of xylose, expression levels of DciA were reduced below the limit of detection (Supplemental Figure S2). Under these conditions, colony formation in the P*xylX*-*dciA* strain was severely compromised (Figure 2B). Further microscopic analysis of the P*xylX*-*dciA* strain showed that loss of *dciA* results in production of extensively elongated cells (Figure 2C,D). These observations were not the result of polar effects on the *dciA*-flanking genes, *CCNA_R0106* (small non-coding RNA) or CCNA_00381 (DNA glycosylase), because colony formation and cell morphology of the DciA-depleted cells were fully restored by introduction of a low copy number plasmid expressing the *dciA* gene, but not a control vector (mean cell lengths of 2.1 ± 0.66 μm and 4.3 ± 3.5 μm, respectively) (Figure 2B–D). These findings support the idea that *dciA* is an essential gene in *C. crescentus*. Moreover, flow cytometric analysis showed that a major fraction of DciA-depleted cells contained one or two chromosomes, and only a small fraction of cells displayed accumulation of more than two chromosomes (Figure 2C), arguing that the essentiality of the *dciA* gene is closely associated with chromosome replication.

### DciA facilitates chromosome replication

**Figure 3.**
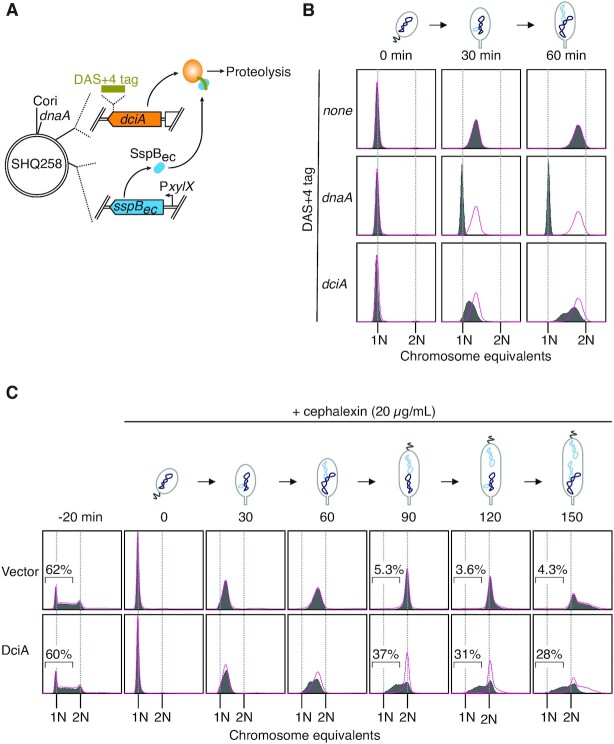
DciA is important for fork progression (**A**) Schematic representation of a DciA-degradable strain (SHQ258), in which the native *dciA* gene is replaced with a *dciA*-*DAS + 4* fusion, and the *E. coli sspB* gene (*SspBec*) is integrated downstream of the xylose dependent promoter (P*xylX*) at the *xylX* locus. Upon induction of SspBec with 0.1% xylose, DAS + 4-tagged DciA is degraded by the ClpXP protease. The relative positions of *Cori*, *dnaA*, *dciA* and *xylX* are indicated on the circular *C. crescentus* genome. (**B**) DNA replication activity in the absence of DicA. SHQ254 (NA1000 *xylX*::*sspB_EC_*) and its derivatives, DnaA-degradable SHQ259 (NA1000 *xylX*::*sspB_EC_ dnaA*::*DAS + 4*) and DciA-degradable SH258 (NA1000 *xylX*::*sspB_EC_ dciA*::*DAS + 4*), cells were grown exponentially in PYE medium supplemented with 0.1% glucose. After addition of 0.1% xylose, cells were further incubated for 20 min at 30°C, followed by cell cycle synchronization. The harvested G1 phase cells were incubated at 30°C in PYE medium supplemented with 0.1% xylose. A portion of the cells was withdrawn at the indicated time, fixed in 70% ethanol, and analyzed by flow cytometry. The DNA profiles of the control SHQ254 cells are colored in red and overlaid with those of SHQ259 and SHQ258. (**C**) DNA replication activity upon overexpression of DciA. Wild-type NA1000 cells harboring pQF (vector) or pQFdciA (DciA) were grown exponentially at 30°C in PYE medium supplemented with tetracycline. After addition of the inducer, cumate (1 μM), cells were further incubated for 20 min at 30°C, followed by cell cycle synchonization. The harvested G1 phase cells were incubated at 30°C for 0–150 min in PYE medium supplemented with 10 μM cumate, 20 μg/ml cephalexin, and tetracycline. A portion of the cells were withdrawn at the indicated time, fixed in 70% ethanol, and analyzed by flow cytometry. The DNA profiles of cells with the vector control are colored in red and overlaid with those of cells with pQFdciA.

To explore the role of DciA in chromosome replication, we performed flow cytometry analysis using synchronized *C. crescentus* cells. We reasoned that if DciA is a component of replication fork proteins, its absence may cause delay in S-phase entry and/or progression. We therefore assessed cellular DNA content over time in a synchronous population of cells. Moreover, to inactivate *dciA* in a timely manner, we made use of an inducible protein degradation system by which a DAS + 4-tagged target protein undergoes rapid proteolysis in a manner that is dependent on the *E. coli* SspB_ec_ adaptor (Figure 3A, Supplemental Figure S2A) (42). In this set up, SspB_ec_ was induced from P*xylX* for 20 min before isolation of synchronized cells. In a control strain without a DAS + 4-tagged protein, the synchronized *C. crescentus* cells expressing SspB_ec_ harbored a single chromosome at the beginning of synchrony and gradually increased DNA content as cells progressed through the cell cycle (Figure 3B). This is consistent with the DNA replication profiles previously reported for wild-type *C. crescentus* cells (43). When a similar analysis was performed in a background with a DAS + 4 tag introduced into the C-terminal coding region of *dciA*, induced degradation of DciA resulted in a substantial delay in S-phase progression (Figure 3B). This suggests that DciA facilitates the activities of the DNA replication forks. Because the delay was evident even at 30 min, the absence of DciA might also dampen the process for DNA replication initiation. However, the observation contrasts with the strong arrest of replication initiation seen in the DnaA-degradable strain; i.e. upon DnaA degradation, nearly all cells harbored a single chromosome even at 60 min after release from synchrony (Figure 3B). Although we do not rule out the possibility that some residual DciA molecules might alleviate the initiation-specific phenotype of the DciA-degradable strain, these data strongly argue that DciA and DnaA play different roles in chromosome replication.

To further investigate the role of *dciA* in chromosome replication, the effects of *dciA* overexpression were assessed using a strain expressing *dciA* from a cumate-dependent promoter on a low-copy number plasmid (pQFdciA). In the presence of the inducer, cumate, the amounts of DciA were increased by 1.3–20 fold in a dose-dependent manner (0.25–2 μM cumate) and reached a plateau (25-fold) at 10 μM cumate (Supplemental Figure S2B). Similar to DciA-depleted cells, increasing levels of DciA resulted in production of elongated cells (Supplemental Figure S3). DNA staining with SYTOX Green fluorescence dye revealed that those elongated cells displayed fewer fluorescent signals compared to the cells with the control vector, suggesting that DNA replication is compromised in cells overexpressing DciA.

To better resolve defects in chromosome replication, we further analyzed cells blocked for cell division with the antibiotic cephalexin, reasoning that cephalexin-treatment allows for additional rounds of chromosome replication in predivisional cells and thus, such over-replicating cells are easily distinguished from cells compromised for replication. As expected, when DNA replication in a synchronous population was monitored under cephalexin treatment, nearly all cells with an empty vector initiated chromosome replication within 30 min and completed it by 90 min (Figure 3C). Subsequently, these cells initiated the next round of chromosome replication, as represented by production of cells with more than two chromosomes at 90 to 150 min (Figure 3C). By contrast, in cells overexpressing DciA with 1 μM cumate (∼10-fold excess amounts of DciA), a large fraction (37%) of cells displayed a substantial delay in chromosome replication at 90 min and 28% of cells failed to complete the first round of chromosome replication after 150 min (Figure 3C). Notably, in these cells, the DNA profile at 30 min was similar to that of the vector control, implying that DciA overexpression has little effect on initiation of DNA replication. Thus, these results indicate that excess amounts of DciA perturb progression, but not initiation of DNA replication, supporting the hypothesis that DciA acts as a replication fork protein to coordinate chromosome replication.

### DciA binds DnaB

**Figure 4.**
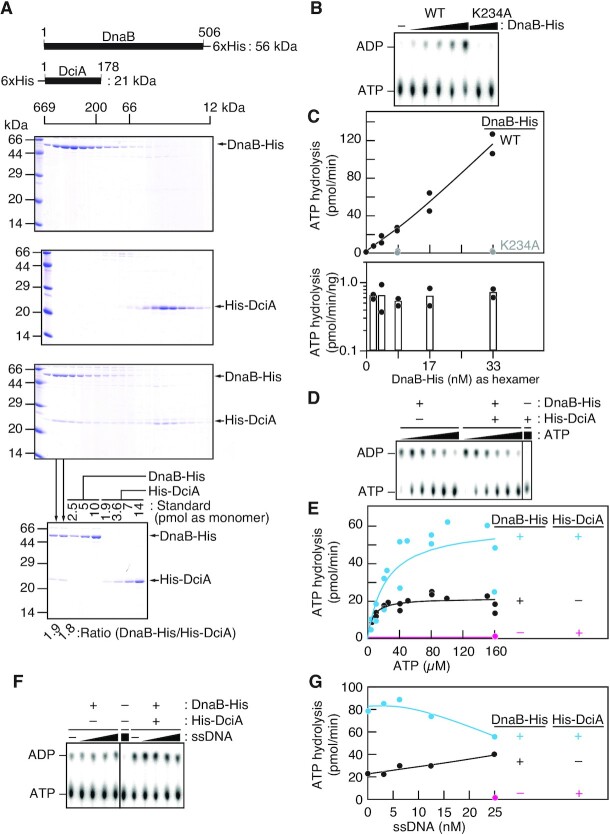
DciA stimulates ATP hydrolysis by DnaB. (**A**) Size exclusion chromatography of DnaB and DciA. DnaB-His (10 nmol; top), His-DciA (25 nmol; middle), or a mixture of both (10 nmol of DnaB-His and 25 nmol of His-DciA; bottom) were separated using a Superdex 200 column. The elution fractions were analyzed by SDS–15% PAGE and Coomassie brilliant blue staining. The elution positions of the molecular weight marker proteins are indicated. To deduce the stoichiometry of DnaB-His per His-DciA in the elution fractions (lanes 2 and 3) for the DnaB-His/His-DciA mixture, the same samples were reanalyzed by SDS–15% PAGE and Coomassie brilliant blue staining (bottom). The indicated amounts of DnaB-His and His-DciA proteins were used as quantitative standard. (**B**–**G**) ATPase assay. [α-^32^P]ATP was incubated with DnaB-His at 30°C for 10 min, followed by thin layer chromatography (TLC). A representative chromatograph (B) and kinetic analysis (C) for a titration of DnaB-His (2.1, 4.2, 8.3, 17, or 33 nM as hexamer) in the presence of 0.1 mM [α-^32^P]ATP were shown. WT, wild type. K234A, an ATPase-dead variant. For panel D and E, ATP hydrolysis of DnaB-His (8.3 nM as hexamer; 50 nM as monomer) was analyzed using TLC at various [α-^32^P]ATP concentrations (5–160 μM) in the presence or absence of His-DciA (100 nM as monomer) (D) and the ATPase rate was fit using Michaelis-Menten kinetics in JMP statistical software (https://www.jmp.com). For panel F and G, ATP hydrolysis was analyzed in buffer containing [α-^32^P]ATP (100 μM), DnaB-His (0 or 8.3 nM as hexamer), HisDciA (0 or 100 nM as monomer), and 76-mer ssDNA (0, 3.1, 6.3, 13 or 25 nM of oligo 764).

To obtain molecular insights into DciA during chromosome replication, we set out to analyze the interaction between DciA and DnaB using size exclusion chromatography on the following purified recombinant proteins: DciA with an N-terminal hexahistidine tag (His-DciA; 21 kDa) and DnaB with a C-terminal hexahistidine tag (DnaB-His; 56 kDa) (Figure 4A). The oligomeric states of His-DciA and DnaB-His were first determined using a Superdex 200 column. His-DciA eluted near a position corresponding to a monomeric state (Figure 4A, Supplemental Figure S4), suggesting that *C. crescentus* DciA is a stable monomer in solution. This observation is consistent with recent reports that a DciA homolog from *V. cholerae* adopts a monomeric state in solution (31). Conversely, DnaB homologs are known to display variety in the oligomeric states ranging from monomeric to dodecameric (18,20,44). Here, DnaB-His eluted at a peak position corresponding to the average molecular weight of a DnaB-His hexamer (330 kDa) (Figure 4A, Supplemental Figure S4). This suggested that the majority of *C. crescentus* DnaB forms a hexameric oligomer, which is reminiscent of the hexameric ring formed by *E. coli* DnaB (DnaB_EC_).

To assess the interaction between DciA and DnaB, a mixture of His-DciA and DnaB-His was analyzed using a Superdex 200 column. In this analysis, the two proteins coeluted earlier than either protein alone, indicating that DciA directly binds DnaB (Figure 4A). The coelution peak corresponded to an average molecular weight of approximately 460 kDa (Supplementary Figure S4) and the stoichiometry of DnaB-His to HisDciA deduced from SDS-PAGE was 1.9 (bottom panel in Figure 4A), which would suggest that each DnaB hexamer binds at least three His-DciA monomers.

### DciA promotes ATP hydrolysis by DnaB

To investigate whether DciA promotes the ATPase activity of DnaB, we performed an *in vitro* ATP hydrolysis assay using pooled peak fractions of DnaB-His from the size exclusion chromatography. In the absence of DciA, ATP hydrolysis was readily observed at various concentrations of wild-type DnaB-His (Figure 4B). As expected, this activity was completely diminished for the DnaB (K234A)-His variant in which the conserved Lys-234 residue of the Walker A motif is substituted with Alanine (Figure 4B). Moreover, the specific activity for ATP hydrolysis by wild-type DnaB-His (200 nM) was comparable to that of *E. coli* DnaB_EC_ reported earlier (16) and remained constant even when low concentrations of DnaB-His (12.5 nM) were used. Given a hexameric form is a prerequisite for the ATPase activity (45), these results argued that the DnaB-His hexamers are substantially stable at submicromolar concentrations.

We next assessed if DciA stimulates the ATPase activity of DnaB. Consistent with the predicted DciA structure, His-DciA per se exhibited no ATPase activity (Figure 4D). Strikingly, when co-incubated with His-DciA, DnaB-His displayed a greater activity in ATP hydrolysis (Figure 4D). Analysis on Michaelis-Menten kinetics revealed that His-DciA increased the maximum velocity for ATP hydrolysis of DnaB-His by ∼3-fold (Figure 4E), indicating that DciA stimulates the ATPase activity of DnaB.

ssDNA was reported to stimulate ATP hydrolysis by *E. coli* DnaB_EC_ (16). Similarly, we found that the presence of 78-mer ssDNA yields a modest increase in the ATPase activity of *Caulobacter* DnaB-His (Figure 4F). This activity was further stimulated by His-DciA when DnaB-His and ssDNA were present in near equimolar concentrations (8.3 nM of DnaB-His as hexamer and 3.3–13 nM of ssDNA) (Figure 4F). Although the effect of His-DciA was less pronounced at relatively high concentrations of ssDNA (25 nM) in excess to DnaB-His, these observations implied that DciA could stimulate ATP hydrolysis of DnaB bound to ssDNA.

### DciA promotes DnaB helicase activity

**Figure 5.**
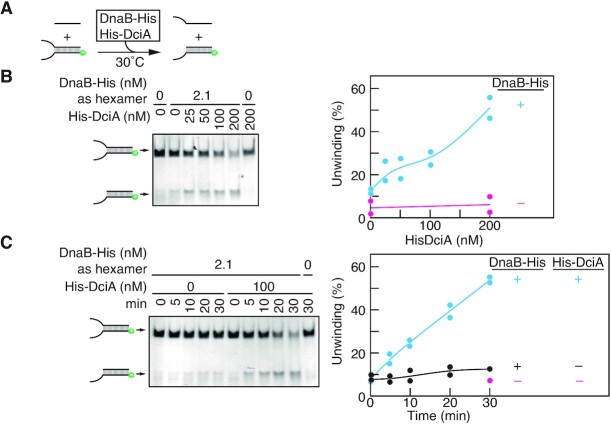
DciA stimulates the DnaB helicase activity DNA helicase assay using a forked DNA substrate. A schematic of the assay is shown (**A**). The bottom strand of the forked DNA substrate is 5′-FAM labelled (green circles) and hybridizes with competitor ssDNA upon separation from the upper strand of the forked DNA substrate. The substrate DNA (12.5 nM) was incubated at 30°C for 0–30 min in buffer containing competitor ssDNA (62.5 nM) and the indicated concentrations of DnaB-His and His-DciA. The products were separated using 6% polyacrylamide gel electrophoresis. Reaction curves and representative gel images for a DciA-titration experiment (**B**) and a time-course experiment (**C**) are shown.

To investigate whether DciA promotes the helicase activity of DnaB, we performed an *in vitro* helicase assay using the forked DNA substrate (22). In this assay, given that the hexameric DnaB ring can thread the 5' ssDNA tail of a forked DNA substrate, subsequent migration of the DnaB hexamer toward the 3′ DNA end results in unwinding of the dsDNA region and the separated bottom strand DNA hybridizes with a short oligonucleotide in the reaction mixture (Figure 5A). As expected, unwinding of the substrate occurred in the presence of DnaB-His hexamer (Figure 5B). Notably, when mixed with His-DciA, the helicase activity of DnaB-His hexamer was markedly increased in a His-DciA dose-dependent manner. Similar results were obtained in a time course experiment (Figure 5C). Thus, these results strongly argue that DciA stimulates the helicase activity of DnaB.

### DciA facilitates DnaB loading onto a single-stranded DNA bubble

**Figure 6.**
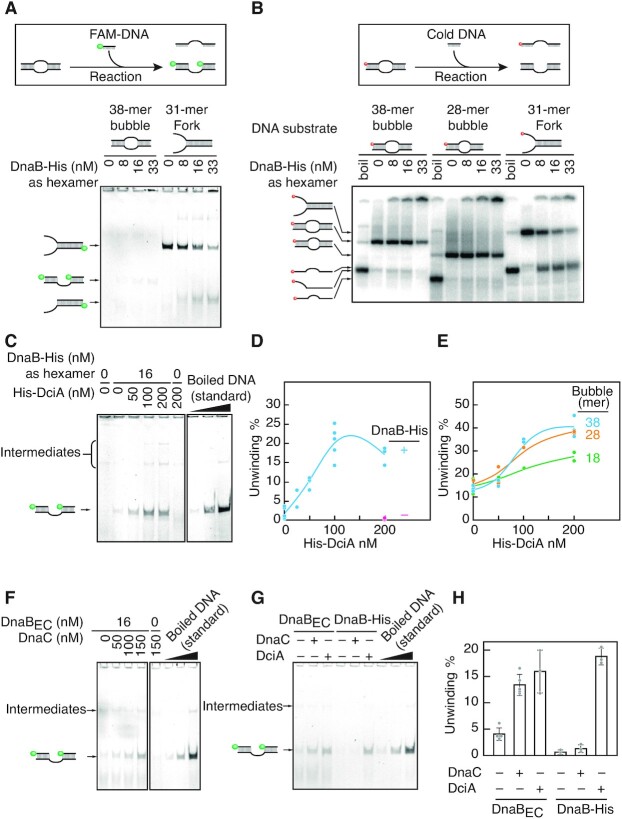
DciA stimulates loading of the DnaB helicase onto the bubble DNA structure (**A**, **B**) A bubble DNA substrate (12.5 nM) was incubated at 30°C for 20 min in buffer containing 5′-FAM labelled competitor ssDNA (62.5 nM) and the indicated concentrations of the recombinant proteins. The products were separated using 9% polyacrylamide gel electrophoresis (A). For panel B, ^32^P-labelled DNA substrate and cold competitor were used instead of cold DNA substrate and 5′-FAM labelled competitor. In both cases, separation of the bubble DNA substrate allows the bottom strand to hybridize with competitor ssDNA. A forked DNA substrate was used as control. A schematic of the assay is shown above the gel image. The 5′-DNA ends labelled by FAM and ^32^P were indicated by green circles and red circles, respectively. (**C**, **D**) His-DciA titration experiments in the presence of DnaB-His (16 nM as hexamer). A representative gel image is shown (C). Scatter plots generated by multiple independent reactions (*n* > 3) are used to draw reaction curves (D). The number of points reflects the exact sample size. Intermediates of the rection were indicated (see *Supplementary Figure S5* for details). (**E**) His-DciA titration experiments with DNA substrates bearing a different bubble size (18, 28 or 38-mer). A representative gel image was shown in *Supplementary Figure S6C*. (**F**) DnaC titration experiments in the presence or absence of *E. coli* DnaB_EC_ (100 nM). (**G**, **H**) The interplay between His-DciA and *E. coli* DnaB_EC_. The activities of DnaB-His and *E. coli* DnaB_EC_ were assessed in the presence of HisDciA or DnaC (100 nM). A representative gel (F) and bar graphs (G) with scatter plots generated by multiple independent reactions (*n* > 3) are shown. The number of points reflects the exact sample size.

We next wished to elucidate the underlying molecular mechanisms by which DciA activates DnaB. By analogy to *E. coli* DnaC and phage lambda P, we hypothesized that DciA facilitates topological DnaB loading onto ssDNA through opening of the DnaB ring. To test this idea, a DnaB loading assay was developed using a replication bubble-mimetic DNA substrate comprising a 38-mer ssDNA bubble flanked by 21 bp duplex DNA regions (Figure 6AB; Supplementary Figure S5). We reasoned that if a DnaB hexamer adopts a closed ring, a single-stranded DNA bubble will necessitate opening of the DnaB ring during the loading process. Consistent with this idea, DnaB-His hexamer alone showed little DNA unwinding activity towards the bubble DNA substrate, but high DnaB-His activity on the forked DNA substrate (Figure 6AB, Supplementary Figure S6A). Strikingly, when co-incubated with His-DciA, DnaB-His hexamer displayed helicase activity on the bubble DNA substrate in a His-DciA dose-dependent manner (Figure 6C–E, Supplementary Figure S6A). Consistent results were obtained in a time course experiment: i.e. while reaction intermediates were slightly more abundant than the full unwinding product within 5 min of incubation, the full unwinding reaction proceeded moderately by further incubation in a His-DciA-dependent manner (Supplementary Figure S6B). These observations are fully consistent with the idea that two DnaB helicases are loaded sequentially onto opposite strands. Although the reactions reached a plateau at 20–25% of unwinding, *E. coli* DnaB_EC_ was similarly recruited to a single stranded DNA bubble in a DnaC-dependent manner, suggesting the validity of this assay (Figure 6F). Moreover, reducing the bubble size from 38-mer to 28-mer largely sustained the ability for His-DciA-dependent recruitment of DnaB-His hexamer whereas the recruitment activity was compromised for a DNA substrate with an 18-mer ssDNA bubble (Figure 6E, Supplementary Figure S6C). This coincides with the earlier report that the *E. coli* DnaB_EC_–DnaC complex encircles 26 bases of ssDNA (17). Together, these results strongly argue that *C. crescentus* DciA switches a DnaB hexamer from its default closed conformation into an open form during helicase loading. Intriguingly, although DnaC did not activate *C. crescentus* DnaB-His hexamer, DciA was capable of loading *E. coli* DnaB_EC_ onto a single stranded DNA bubble (Figure 6G, H). These differences might suggest that DciA and DnaC evolved independently to converge on a similar loading mechanism (see discussion).

### DnaB binds a C-terminal extension of DciA

**Figure 7.**
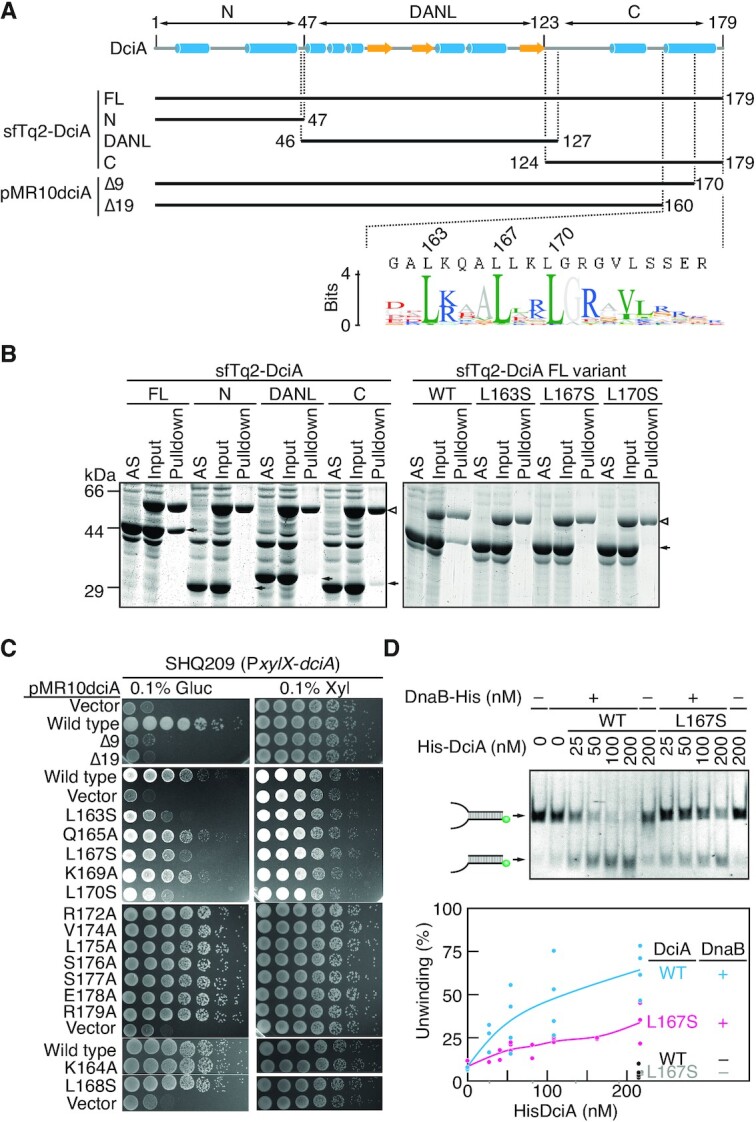
The DciA C-terminus operates DnaB (**A**) Schematic representation of DciA truncation constructs. sfTq2 proteins fused to the N-terminus of full-length DciA (sfTq2-DciA), or the truncated versions (N, 1–47 aa; DANL, 46–127 aa; C, 124–179 aa) were used for the pulldown assays. For plasmid complementation assays, pMR10-dciA derivatives carrying the *dciA* allele (Δ9, 1–170 aa; Δ19, 1–160 aa) were used. The schematic shows the predicted secondary structures (alpha helices in blue and beta strands in orange). Multiple sequence alignments were created by Protein BLAST search for DciA and the top 500 sequence data were used to generate Weblogo representation (66). (**B**) *E. coli* crude extracts with and without sfTq2-DciA were incubated in the presence (+) or absence (–) of DnaB-His, followed by pulldown using Ni-conjugated magnetic beads. After washing, materials retained on the beads were analyzed using SDS–12% PAGE and Coomassie Brilliant Blue staining. AS, ammonium sulfate-precipitated sfTq2-DciA; Input, a mixture of AS and DnaB-His; Pulldown, an elution fraction. (**C**) Plasmid complementation test. pMR10-HAdciA derivatives (Δ9 and Δ19) or pMR10dciA derivatives with the indicated *dciA* allele were analyzed as described in Figure 2D. (**D**) Helicase assay. Wild-type His-DciA or His-DciA(L167S) were analyzed as described in Figure 5. Reaction curves and representative gel images for a DciA-titration experiment are shown.

Finally, to gain molecular insights into DciA-mediated DnaB loading, we carried out pulldown experiments to determine the functional domain of DciA responsible for the DnaB interaction. Based on the predicted *C. crescentus* DciA structure generated by AlphaFold (46), DciA was subdivided into three domains: the central DANL domain (amino acids 47–123), the N-terminal extension (amino acids 1–46), and the C-terminal extension (amino acids 124–179) (Figure 1C). Crude extracts were generated from *E. coli* cells expressing a superfolder mTurquoise 2 fluorescent protein (sfTq2 fused to the N terminus of full-length DciA (sfTq2-DciA FL) or each of the three domains (Figure 7A). When DnaB-His was incubated with the extracts containing sfTq2-DciA FL, we observed coelution of sfTq2-DciA FL with DnaB-His, suggesting an interaction between the two proteins (Figure 7B). Also, sfTq2-DciA FL was pulled down with *E. coli* His-DnaB_EC_ (Supplementary Figure S7), consistent with the result for the helicase assay (Figure 6G-H). Notably, we found that the sfTq2-tagged C-terminal extension retained a basal affinity for DnaB-His. By contrast, neither the N-terminal extension nor the DANL domain supported DnaB binding in our pulldown assay (Figure 7B). Thus, the DciA C-terminus includes a functional motif that interacts with DnaB. In agreement with this, a plasmid complementation assay revealed that the DciA variants lacking the C-terminal residues spanning 170–179 are defective *in vivo* (Figure 7C). A further in-depth analysis using DciA variants revealed that the complementation activity was also compromised by a single amino acid substitution for the conserved leucine residues at positions 163, 167 or 170 (Figure 7A–C). Western blot analyses showed that protein abundance of DciA L167S and L170S variants were comparable to that of wild-type DciA (Supplementary Figure S2C), arguing that the DciA Leu167 and Leu170 residues play specific roles for the DciA function *in vivo*. Also, the DciA Leu163 residue might be involved in DciA stability as the subcellular DciA L163S levels were reduced. Together, these observations are consistent with the idea that the specific interaction of DnaB with the C-terminus of DciA is essential for chromosome replication.

To corroborate the idea that the conserved leucine residues at the DciA C-terminus constitute a binding site for DnaB, we carried out the pulldown assays using sfTq2-DciA FL variants (L163S, L167S, L167S). Unlike wild-type sfTq2-DciA FL, these variants severely reduced the affinity for DnaB-His (Figure 7B). Moreover, focusing on the DciA Leu167 residue, we purified His-DciA (L167S) protein and tested the activity in the helicase assay. As shown in Figure 7D, His-DciA (L167S) was less active in stimulation of the DnaB helicase activity. Together, these data suggest that conserved leucine residues at the DciA C-terminus operate DnaB through direct interaction.

## DISCUSSION

Loading of a replicative helicase is a fundamental process of chromosomal replication required to separate duplex DNA into the template single strands. In this study, we investigated the molecular mechanisms of the replicative DnaB helicase and its interacting partner, DciA, in *C. crescentus*. While homologs of these proteins are highly conserved among bacterial species, it has been difficult to distinguish between their functions in replication initiation and elongation, and the molecular mechanisms underlying the DnaB-DciA system remain unclear. Here, we provide good evidence that the *C. crescentus* DciA acts as a replication fork protein to render DnaB competent for topological loading onto replication forks. We found that *C. crescentus* DnaB forms a homohexamer that is incapable of self-loading onto ssDNA unless a free ssDNA end is present for its threading, consistent with the idea that the DnaB hexamer takes on a closed ring-shaped structure as the default state. Similar results were reported using a DnaB homolog from *Vibrio cholerae* that expresses cognate DciA homolog (31). Notably, we demonstrate that in the presence of *C. crescentus* DciA, the DnaB hexamer gains topological loading ability, executing strand separation on the single-stranded DNA bubble. Congruently, *in vivo*, *C. crescentus* cells required DciA for normal progression of the replication fork. Taken together, these data lead us to propose that DciA acts as a molecular switch to shift the closed DnaB hexamer into an open structure that encircles the single-strand, thereby resulting in helicase loading onto the replication forks. In this context, it is inferable that in cells overexpressing DciA, excess DciA might reverse the loading reaction, thereby causing compromised DNA replication. A potential involvement of the reversed reaction by the helicase loader may be applicable to the observation that *E. coli* cells overexpressing DnaC inhibit DNA replication (47). Given that DnaB and DciA homologs are conserved in most eubacteria including the pathogenic *Pseudomonas aeruginosa* and *Mycobacterium tuberculosis*, our findings imply a common mechanism underlying chromosomal replication governed by the DciA-DnaB module. Importantly, the ring opening mechanism has been previously reported for DciA-unrelated DnaB modules, i.e. the *E. coli* DnaC and phage lambda P loaders (17,21,27). Thus, our findings expand the understanding of general principles for helicase loading.

The finding that the C-terminal extension of the DANL domain of DciA contains conserved leucine residues that would constitute a primary binding site for DnaB, provides novel insights into the molecular mechanism of the DciA–DnaB module. Recent cryogenic electron microscopy and crystal structure analyses of the helicase loaders demonstrate that both *E. coli* DnaC and phage lambda P loaders interact with the exterior surface of the DnaB hexameric ring (Figure 1, Supplementary Figure S1) (17,21,27). Despite little or no structural homology, these two different helicase loaders exemplify a common strategy in which their interference with the exterior surface of the DnaB hexameric ring likely distorts the closed ring to switch it to an open structure. Therefore, it is reasonable to speculate that *C. crescentus* DciA also employs a similar strategy by making use of its C-terminal extension to interfere with the exterior surface of the cognate DnaB hexameric ring. In this context, we hypothesize that the DANL domain of DciA would assist in DciA binding to DnaB. The DANL domain displays structural homology to the *E. coli* DnaA N-terminal domain (30,31). We previously reported that the N-terminal Phe46 residue of *E. coli* DnaA specifically binds to the Leu160 residue of the cognate DnaB, which resides on the exterior surface region (22,48,49). Furthermore, a truncated *M. tuberculosis* DciA polypeptide that contains the DANL domain was reported to interact with the cognate DnaB (30). Taken together, we propose that both the DANL domain and the C-terminal extension of *C. crescentus* DciA cooperate in helicase loading through their interactions with the exterior surface of the cognate DnaB hexameric ring. In the case of *E. coli* DnaC, the C-terminal AAA + domain displays ssDNA-dependent ATPase activity, allowing the DnaB_EC_ helicase to take on the closed structure (17,23). It will be important to address whether similar mechanisms regulate topological isomerization between the open and closed states of the DnaB ring in the DnaB-DciA system in future studies.

A predicted *C. crescentus* DciA structure generated by AlphaFold implies that the C-terminal extension comprises two alpha helices and a preceding unstructured linker connected to the DANL domain (Figure 1). These structural features are not similar to those of either *E. coli* DnaC or phage lambda P, indicating that this be an example of molecular mimicry resulting from convergent evolution, as proposed recently to account for the relationship between *E. coli* DnaC and phage lambda P (50). In support of this notion, the *E. coli* DnaB residues responsible for interaction with *E. coli* DnaA/DnaC or phage lambda P are moderately or poorly conserved in DnaB homologs from *dciA*-containing species (Supplementary Figure S1). This suggests that these positions may have been altered during the domestication of different helicase loaders. Similarly, the position corresponding to Ser296 of *V. cholerae* DciA, which has previously been called the determinant residue based on its conservation in DnaB homologs from *dciA*-containing Enterobacteriales (31), displays considerable variation in the *dciA*-containing non-Enterobacteriales.

The DNA replication analysis performed in this study using a synchronous population highlights the predominant role of DciA during DNA replication elongation. Using asynchronous cell populations, the DciA homolog (DciA_PA_) from *P. aeruginosa* was previously implicated in the early stage of chromosome replication; however, it did not clearly differentiate between a role for DciA in replication initiation and elongation (29). Indeed, the previous study using a *P. aeruginosa* Δ*dciA_PA_* mutant strain harboring a complementary *dciA_PA_* plasmid with a thermosensitive origin of replication, revealed that a partial loss of the *dciA_PA_* plasmid at the non-permissive temperature results in production of cells with reduced cellular DNA content and moderate arrest in the early stage of chromosome replication. Similarly, the use of a single-strand-specific chemical modification demonstrated further that formation of a replication bubble at the origin of replication was more pronounced at the non-permissive temperature, implying that some of the Δ*dciA_PA_* cells paused DNA replication near or within the origin. These observations are fully consistent with the idea that DciA_PA_ is important for initiation, elongation, or both. Here we demonstrate that cells devoid of DciA continue to initiate DNA replication, but delay elongation of chromosome replication, which strongly argues for a role for DciA in the maintenance of replication forks. The translocating DnaB helicases could fall off the chromosome upon encountering physical obstacles such as RNA polymerases and various DNA binding proteins. Therefore, completion of DNA synthesis necessitates DnaB re-loading onto the stalled replication forks. In *E. coli*, this maintenance is ensured exclusively by the DnaC loader with the aid of primosomal proteins including PriA, PriB, PriC and DnaT (13,14,51). Of these, the PriA homologs are widely conserved in diverse bacterial species including *C. crescentus* (52). Thus, it is possible that DciA acts in concert with PriA to maintain fork progression. Intriguingly, the gene flanking *dciA* encodes a base excision repair MutY homolog and this is seen in diverse alphaproteobacterial species. Hence, these findings also support the idea that DciA operates in the maintenance of replication forks. In living *Caulobacter* cells, DnaB-YFP fusion is reported to form a discrete focus at replication forks (53). Thus, it is conceivable that DnaB loading is also facilitated through an unknown mechanism by which local concentrations of DnaB increase near replication forks.

The finding that DciA stimulates the ATPase activity of DnaB contrasts with the previous reports that *E. coli* DnaC as well as the lambda P protein inhibits the ATPase activity of *E. coli* DnaB_EC_ (54–56). These observations are consistent with the idea that the DciA-DnaB system is mechanistically differentiated from the *E. coli* DnaC-DnaB_EC_ or the lambda P-DnaB_EC_ system. Structural analyses for the *E. coli* DnaC–DnaB_EC_ complex suggest that interference of ATP hydrolysis by DnaC imposes an ADP-bound, ring-opened configuration on DnaB_EC_, thereby stimulating the loading reaction (17). Subsequent DNA translocation of DnaB_EC_, which is dependent on the ATPase activity, thus requires dissociation of DnaC from the complex (23). Our data suggest that interference of the helicase's ATPase activity is dispensable for the DciA-DnaB system. The activation of DnaB ATPase by the accessory subunit is reminiscent of the eukaryotic replicative MCM helicase, the ATPase activity of which is activated by Cdc45 and GINS complexes(57). We speculate that the equilibrium between open and closed configuration of the DnaB-DciA complex is more poised toward the closed state that favors ATP hydrolysis. This scenario also coincides with the idea that, in contrast to DnaC, the DnaB-DciA complex remains associated in a closed-ring configuration after loading reaction to facilitate DNA translocation through enhanced ATP hydrolysis. Alternatively, the ring-opened state of the DnaB-DciA complex might be competent to hydrolyze ATP through adoption of a yet uncharacterized configuration. Testing these scenarios is an important challenge for future work. The *C. crescentus* DnaB-DciA system also contrasts with the helicase operation system from *B. subtilis*. The *B. subtilis* replicative helicase (BsDnaC) takes on various oligomeric states and exists preferentially as a monomer in solution. Upon biding to DnaI, the regulatory subunit belonging to the AAA + family, BsDnaC monomers are assembled into a hexameric ring around DNA, thereby exhibiting the helicase activity (45). Because the preformed BsDnaC hexamer is no longer activated by DnaI (45), it has been proposed that making a hexameric helicase ring, rather than breaking it, underlies helicase loading of this bacterium. This ring making mechanism does not coincide with the *C. crescentus* DnaB-DciA system in that *C. crescentus* DnaB forms a stable hexamer and that the pre-formed DnaB hexamer is activated by DciA.

It remains unclear how DciA contributes to initiation of chromosome replication. We do not fully rule out DciA playing a specific role at the origin, although a G1 cell cycle arrest, a hallmark of replication initiation defects, was not observed in cells devoid of DciA in our experimental conditions. Alternatively, it is tempting to speculate that an as-of-yet unknown protein factor may operate at the origin. In some Gram-positive bacteria including *B. subtilis*, primosomal DnaB, DnaD, and DnaI proteins comprise a helicase loader complex at the origin, while *Helicobacter pylori* employs the unique Hp0897 protein to operate the cognate replicative helicase during replication initiation (58–60). Although no homologous proteins corresponding to the *B. subtilis* or *H. pylori* helicase operators have been annotated in *C. crescentus*, it is possible that in the course of evolution, this organism acquired structural homologs of these proteins with little sequence similarity.

## DATA AVAILABILITY

The data supporting the findings of this study are available from the corresponding author upon reasonable request.

## Supplementary Material

gkac1146_Supplemental_File
