# Supplementary material for: The Caulobacter crescentus DciA promotes chromosome replication through topological loading of the DnaB replicative helicase at replication forks
Source: Nucleic Acids Res. 2022 Dec 9;50(22):12896–912. doi: 10.1093/nar/gkac1146 (PMC9825169; doi:10.1093/nar/gkac1146)
Supplement: gkac1146_Supplemental_File [file gkac1146_supplemental_file.docx]

**Supplemental material**

**Bacterial strains**

All *C. crescentus* strains used in this study are derivatives of the wild-type NA1000 strain. SHQ209 and SHQ254 were generated by double homologous recombination using pNPTS-PxyldciA and pNPTS-Pxyl-sspB, respectively. SHQ258 and SHQ259 were generated by plasmid integration using pMCS4dciAssrA and pMCS4dnaAssrA, respectively.

**Plasmid construction**

To generate pNPTS-PxyldciA, a 6.5-kb vector DNA fragment was amplified by PCR using pNPTS3FdciA and primers 575/576. A 0.74 kb DNA fragment including the PxylX was amplified by PCR using pNPTSPxylDivKpleD and primers 577/578. After digestion with NdeI and KpnI, a 0.35 kb insert DNA fragment was excised and ligated to the vector DNA. To construct pNPTS3FdciA, a 0.69 kb DNA fragment including the region upstream of *dciA* and 3xFLAG tag was amplified by PCR using NA1000 and primers 307/362. Also, a 0.59 kb DNA fragment including the dciA ORF was amplified by PCR using NA1000 and primers 311/312. These two DNA fragments were integrated at the SpeI-EcoRI sites of pNPTS138.

To generate pMR20dciA, a 0.67-kb insert DNA fragment was amplified by PCR using NA1000 and primers 392/393. After digestion with HindIII and KpnI, the product was ligated to the HindIII-KpnI fragment of pMR20.

To generate pMR10HAdciA, a 1.0-kb insert DNA fragment was generated by overlap extension PCR. First, a 614-bp DNA fragment was amplified by PCR using NA1000 and primers 291/292. Second, a 454 bp DNA fragment was amplified by PCR using NA1000 and primers 293/294. These fragments were combined by overlap extension PCR using primers 291/294. After digestion with HindIII and EcoRI, the products were ligated to the HindIII-EcoRI fragment of pMR10. To generate a pMR10HAdciA derivative with the *dciA∆9* allele, the BamHI-HindIII fragment (0.56-kb) was excised from pMR10HAdciA and replaced by the BamHI-HindIII fragment generated by PCR using NA1000 and primers 738/739, resulting in pMR10HAdciA170. Similarly, pMR10HAdciA160 with the *dciA∆19* allele was generated using primers 738/740, instead of 738/739. For construction of pMR10-dciA and its derivatives with a dciA allele (R171A, V173A, L174A, S175A, S176A, E177A, or R178A), a 1.0-kb insert DNA fragment was amplified by PCR using NA1000, a forward primer 294 and a reverse primer (291 for WT; 1393 for R171A; 1394 for V173A; 1395 for L174A; 1396 for S175A; 1397 for S176A; 1398 for E177A; 1399 for R178A). The pMR10-dciA derivatives with a dciA allele (L163S, K164A, Q165A, L167S, L168S, K169A, L170S) were constructed by invert PCR. First, a 4.2 kb DNA fragment was generated by PCR using pMR10-dciA and primers 1416/708. Second, a 5.5 kb DNA fragment was generated by PCR using pMR10-dciA, a forward primer 709, and a mutagenic primer (1417 for L163S; 1418 for K164A; 1419 for Q165A; 1420 for L167S; 1421 for L168S; 1422 for K169A; 1423 for L170S). After digestion with BamHI, these fragments were ligated.

To generate pMCS4dciAssrA, a 0.52-kb DNA fragment containing a 5’ part of the *dciA*-coding region without a stop codon was amplified by PCR using NA1000 and primers 196/197. A short cassette encoding the ssrA-DAS+4 tag was generated by annealing synthetic oligonucleotides 782/783. These products were introduced into the XhoI-EcoRI site of pMCS-4.

To generate pMCS4dnaAssrA, a 0.68-kb DNA fragment containing a 5’ part of the *dciA*-coding region without a stop codon was amplified by PCR using NA1000 and primers 102/103. A short cassette encoding the ssrA-DAS+4 tag was generated by annealing synthetic oligonucleotides 784/785. These products were introduced into the SacI-EcoRI site of pMCS-4.

To generate pNPTS-Pxyl-sspB, a 0.52 kb DNA fragment including the *sspB_ec_* coding region was amplified by PCR using *E. coli* MG1655 and primers 780/781, digested with NdeI and EcoRI, and ligated to the NdeI-EcoRI fragment of pNPTSxylXlacImCherry. pNPTSxylXlacImCherry was generated by overlap extension PCR. First, a 0.70-kb DNA fragment containing the Pxyl promoter was amplified by PCR using NA1000 and primers 467/468. Second, a 1.8-kb DNA fragment was amplified by PCR using pLacQFlacImCherry and primers 471/472. Third, a 0.74-kb DNA fragment containing the region downstream of the Pxyl promoter was amplified by PCR using NA1000 and primers 471/472. These fragments were combined by overlap extension PCR using primers 468/472. After digestion with SpeI and SphI, the products were ligated to the SpeI–SphI fragment of pNPTS138.

To generate pQF00380, a 0.55-kb DNA fragment containing the *CCNA_00380* coding region was amplified by PCR using NA1000 and primers 191 and 192, digested with HindIII and SacI, and ligated to the HindIII-SacI fragment of pQF.

To generate pET21aHisdciA, a 0.6 kb DNA fragment was amplified by PCR using NA1000 and primers 808/606, digested with XbaI and HindIII, and ligated to the XbaI-HindIII fragment of pET21a. For pET21aHisdciA(L167S), pMR10dciA(L167S), instead of NA1000, was used as PCR template.

To generate pET28aCCNA01737cHis, a 1.5 kb DNA fragment containing the *CCNA_01736 (dnaB)*-coding region was amplified by PCR using NA1000 and primers 607/608, digested with NdeI and NotI, and ligated to the 5.3 kb DNA of pET28a. For construction of the pET28aCCNA01737cHis allele with the *dnaB* *K234A* allele, a 0.70-kb DNA fragment was amplified by PCR using NA1000 and primers 607/1100. Also, a 0.86-kb DNA fragment was amplified by PCR using NA1000 and primers 1101/645. These fragments were combined by overlap extension PCR using primers 607/645. After digestion with NdeI and NotI, the products were ligated to the NdeI–NotI fragment of pET28a, resulting in pET28aDnaBcHis(K234A).

To generate pET28-sfTq2dciA(WT)-d, a 0.76-kb DNA fragment containing the sfTq2-coding region was amplified by PCR using sfTq2 and primers 425/426. A 0.60-kb DNA fragment containing the dciA-coding region was amplified by PCR using NA1000 and primers 1169/770. These two fragments were introduced into the XbaI-NdeI site of pET28aCCNA01737cHis, resulting in pET28-sfTq2dciA(WT)-dnaBcHis. Then, the *dnaB-chis*-coding region was excised from pET28-sfTq2dciA(WT)-dnaBcHis by NdeI and XhoI digestion, and the remaining fragment was blunted and self-ligated, resulting in pET28-sfTq2dciA(WT)-d. For the pET28-sfTq2dciA(WT)-d derivatives expressing sfTq2DciA variants (L163S, L167S, or L170S), the pMR10dciA derivatives with the corresponding *dciA* allele, instead of NA1000, were used as PCR template. The pET28-sfTq2dciA(WT)-d derivatives expressing truncated sfTq2DciA variants were generated similarly using mutagenic primers (1169/1170 for pETsfTq2dciA(2-47)-d; 1171/1172 for pETsfTq2dciA(46-127)-d; 1173/770 for pETsfTq2dciA(124-179)-d), instead of 1169/770.

**Proteins**

To purify DnaB-His, Rosetta™ 2(DE3) (Novagen) cells harboring pET28aCCNA01737cHis were grown exponentially in 2.5 L of PY medium (10 g/L peptone, 5 g/L yeast extract, and 5 g/L sodium chloride) supplemented with kanamycin and chloramphenicol, and expression of DnaB-His proteins was induced at 37˚C for 1 h by addition of 1 mM isopropyl-β-D-thiogalactoside. The induced cells were harvested by centrifugation and resuspended in buffer A (25 mM Tris-HCl [pH 7.5], 300 mM sodium chloride, 0.1 mM ATP, and 5% glycerol) supplemented with 5 mM imidazole, 0.1% Triton X-100, and 0.2 mg/mL lysozyme. The cell suspension was incubated on ice for 30 min, sonicated briefly, frozen in liquid nitrogen, and thawed to lyse the cells. After ultracentrifugation (48000 rpm, 20 min), the supernatant was loaded onto a Ni Sepharose 6 Fast Flow column (2 mL) equilibrated with buffer A containing 5 mM imidazole. After washing with buffer A containing 40 mM imidazole, DnaB-His proteins retained on the column were eluted in buffer A containing 500 mM imidazole. The peak fraction was further purified by size exclusion chromatography using Superdex 200 (24 mL) equilibrated with SEC buffer (25 mM Tris HCl [pH 7.5], 300 mM NaCl, 20% glycerol, 0.1 mM ATP, 5 mM magnesium chloride). Fractions corresponding to a DnaB hexamer were pooled and used for the biochemical assays.

To purify His-DciA proteins, Rosetta™ 2(DE3) (Novagen) cells harboring pET21aHisdciA were grown exponentially in 1 L of LB medium supplemented with ampicillin and chloramphenicol and expression of His-DciA proteins was induced at 30˚C for 1 h by addition of 1 mM isopropyl-β-D-thiogalactoside. The His-DciA proteins were purified as described for DnaB-His purification.

**Supplementary Figure S1. Sequence analysis of DnaB homologs**

Multiple sequence alignment of DnaB homologs from *dciA*-containing organisms (CC, *Caulobacter crescentus*; VC, *Vibrio cholerae*; MT, *Mycobacterium tuberculosis*; PA, *Pseudomonas aeruginosa*) and a *dnaC*-containing organism (EC, *Escherichia coli*). Arrows indicate *E. coli* DnaB residues that interact with *E. coli* DnaA (red), *E. coli* DnaC (blue), and phage lambda P (green).


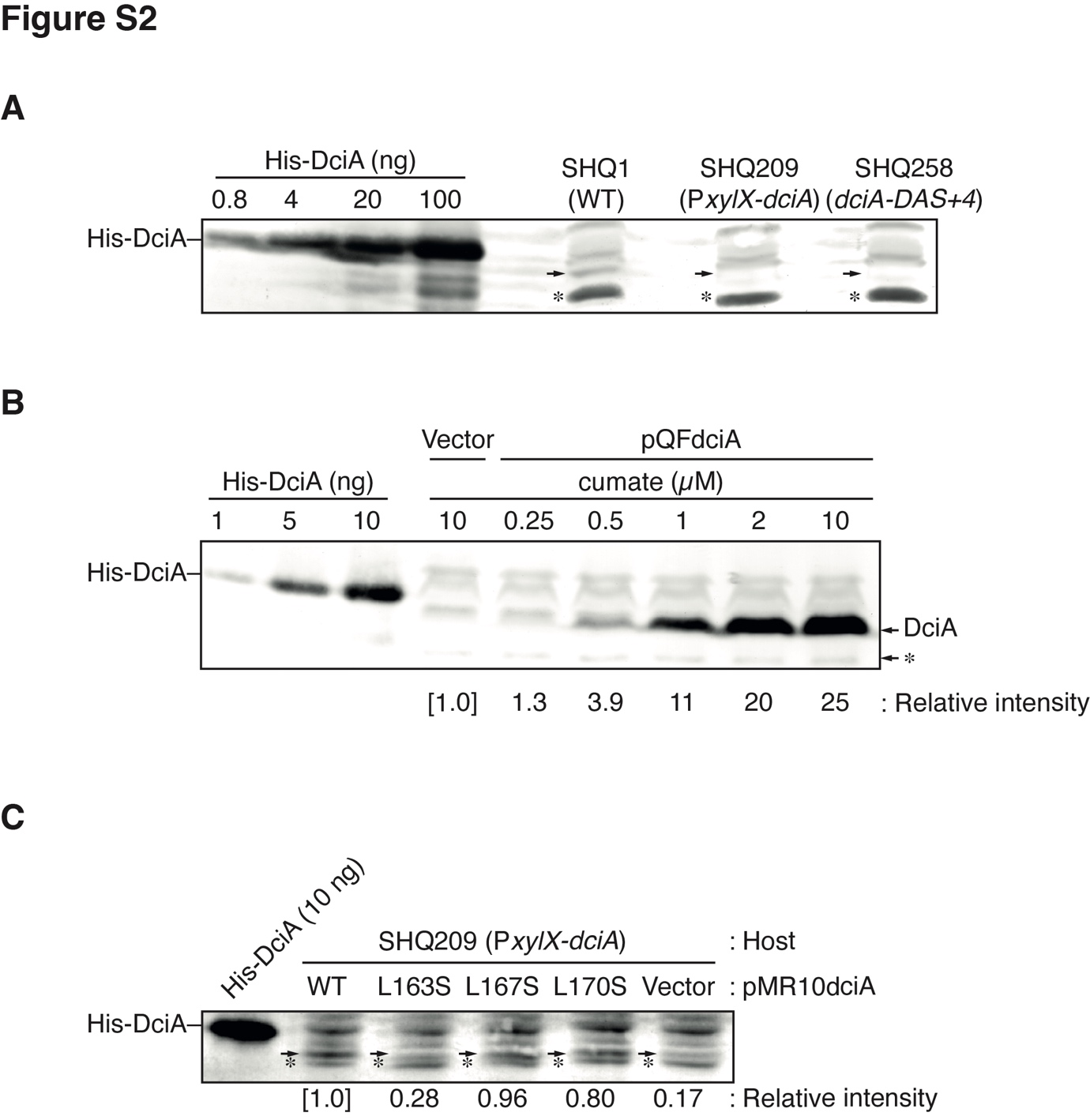


**Supplementary Figure S2. Western blotting of DciA expression**

DciA levels in the indicated strains (SHQ1, SHQ209, SHQ258) (A) and expression of DciA from a pQF vector plasmid (B) or a pMR10 plasmid (C) were analyzed by western blotting using anti-DciA antibody (1:1000 dilution). Purified His-DciA proteins were used to draw a standard curve. A protein that cross-reacts with the anti-DciA antibody is indicated by asterisks. For SHQ1 and SHQ209, cells grown for 6 h in PYE medium supplemented with glucose were analyzed For SHQ258, cells grown for 1 h in PYE medium supplemented with xylose were analyzed. For panel *B*, SHQ1 cells harboring pQF or pQFdicA were grown exponentially at 30˚C in PYE medium supplemented with tetracycline. After 1 h induction with cumate, cells were harvested for the analysis. The band intensity of DciA relative to that in the vector control was shown below the gel image. For panel *C*, SHQ209 cells harboring pMR10 (Vector), pMR10dciA(WT), pMR10dciA(L163S), pMR10dciA(L167S) or pMR10dciA(L170S) were grown for 6 h in PYE medium supplemented with glucose, followed by the analysis. The band intensity of DciA relative to that for pMR10dciA (WT) was shown below the gel image.


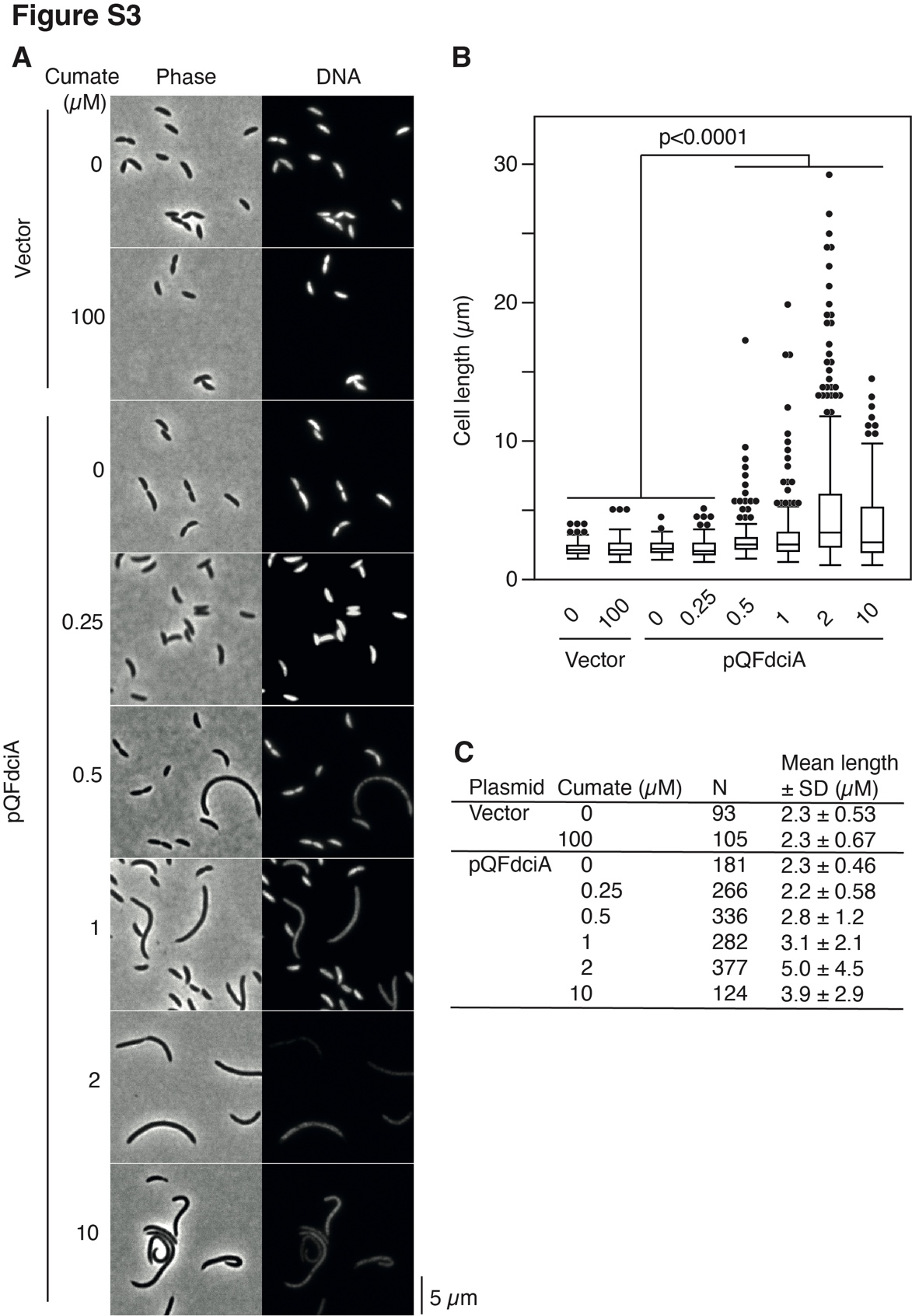


**Supplementary Figure S3. Microscopic analyses of cells overexpressing DciA**

SHQ1 harboring pQF (Vector) or pQFdciA were grown for 9 h in PYE medium supplemented with tetracycline and the indicated concentrations of cumate, followed by fixation in 70% ethanol. After DNA staining with SYTOX green (Thermo), phase-contrast (Phase) and fluorescent (DNA) images were taken using fluorescence microscopy (A). The distributions of cell lengths are shown using a box plot (B). The *P* value was calculated using the Mann-Whitney-Wilcoxon test. Also, the number of cells analyzed (N) and mean cell length with standard deviation (SD) are indicated (C).


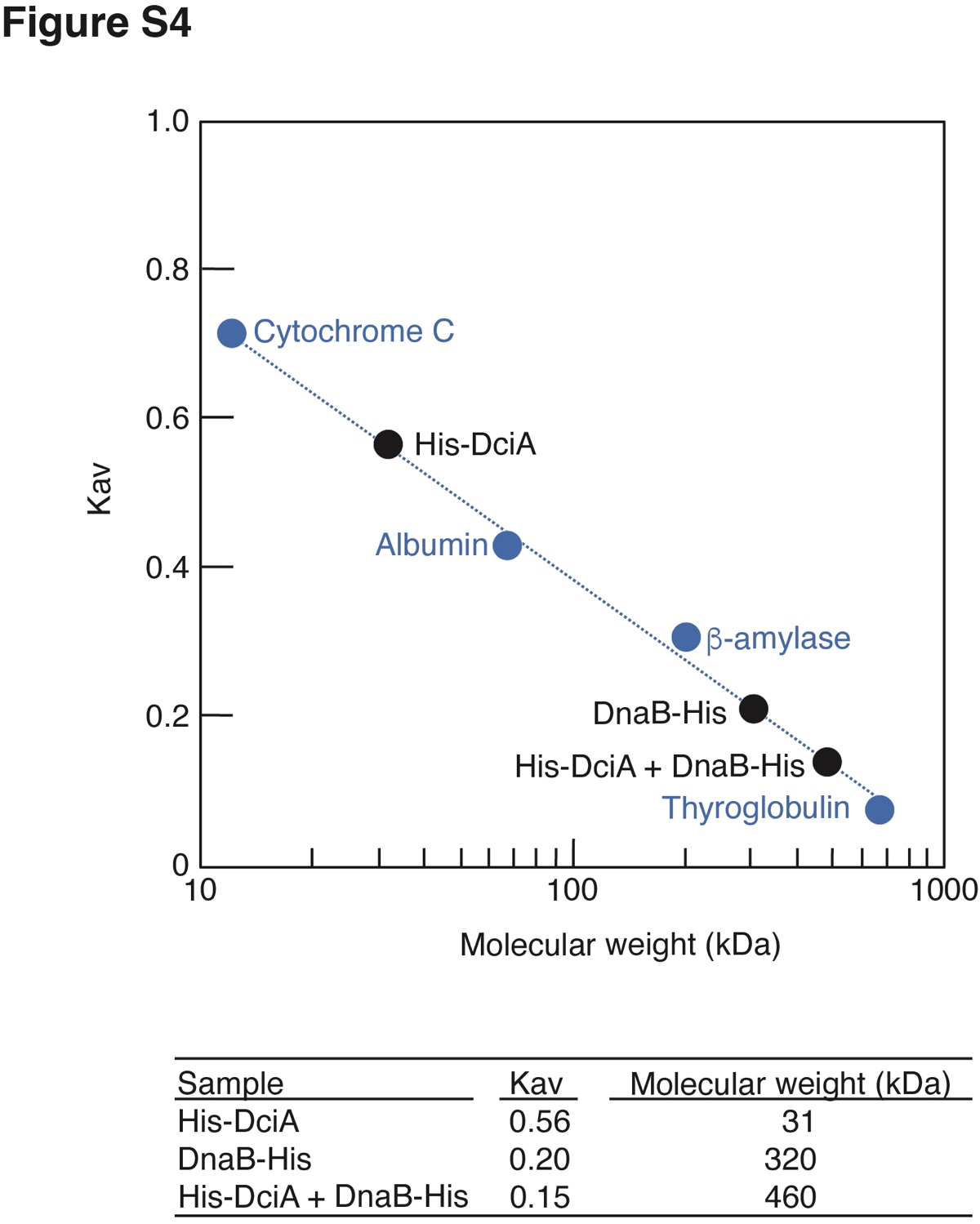


**Supplementary Figure S4. The profiles of elution peaks in size exclusion chromatography**

The molecular weights for His-DciA, DnaB-His, and a complex of His-DciA and DnaB-His were deduced from the elution peaks on size exclusion chromatography shown in *Figure 4A*. Calibration curve was generated by plotting Kav (partition coefficient) against the molecular weights of marker proteins (Thyroglobulin 670 kDa, β-Amylase 200 kDa, Albumin 66 kDa, and Cytochrome C 12 kDa). The molecular weight of DnaB-His (320 kDa) coincides well with the theoretical molecular weight of a DnaB-His hexamer (330 kDa). The molecular weight of His-DciA was slightly larger than the theoretical value for His-DciA monomer (21 kDa), the difference between which could reflect the shape of the DciA molecule which is predicted to contain N- and C-terminal extensions as shown in *Figure 1C*.


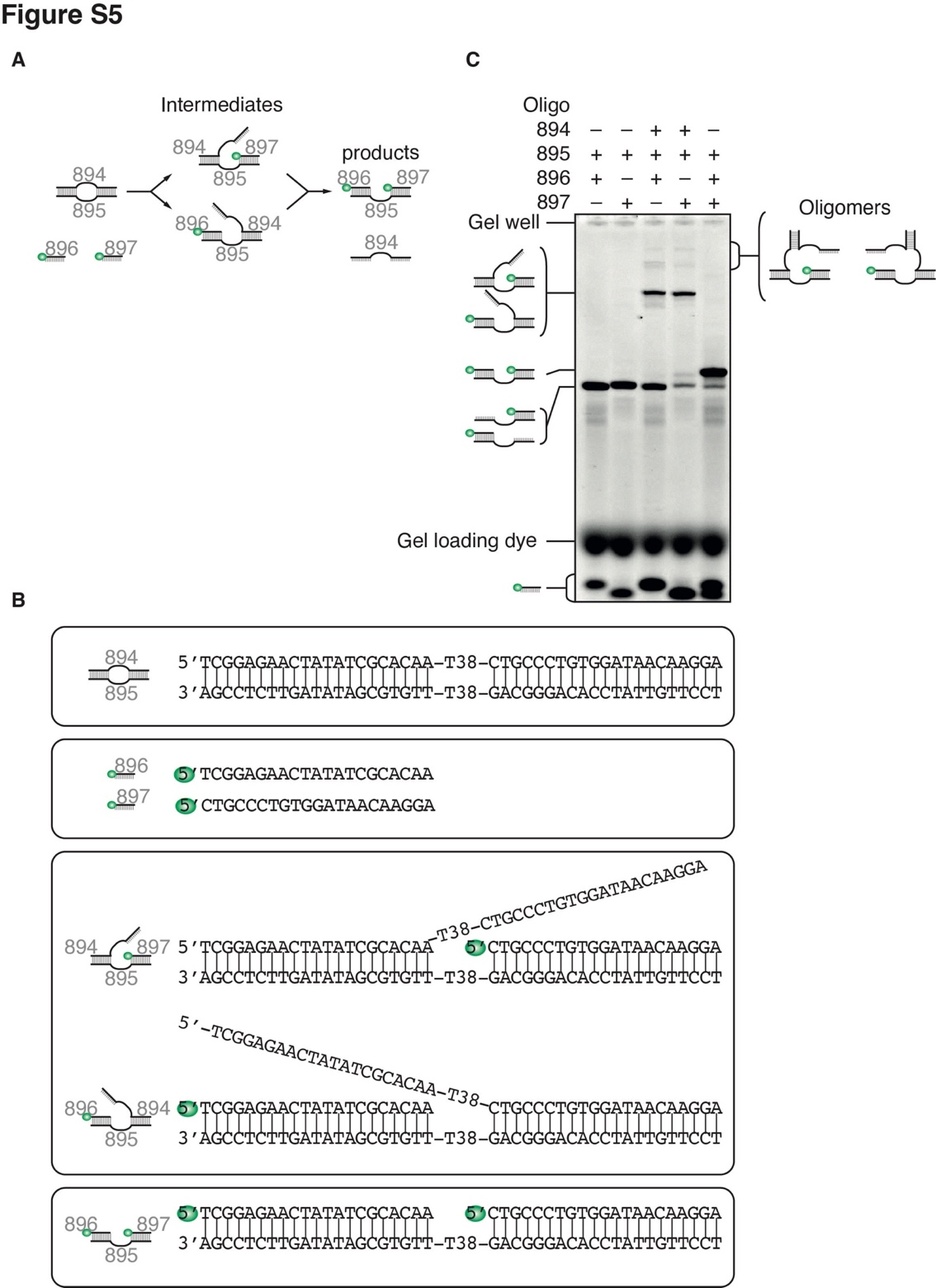


**Supplementary Figure S5. DNA structures related to *Figure 6***

(A-B) Schematic illustration of the DNA helicase assay using a bubble DNA substrate. Conceivable reaction intermediates and products are shown (A). Green circles indicate FAM labelling. The numbers indicate the oligonucleotides listed in *Table 3*. The DNA sequences are shown (B). T38, 38-mer polythymidine.

(C) Gel electrophoresis. Equimolar amounts of the indicated oligonucleotides (0.17 µM) were mixed in buffer (20 mM Tris HCl [pH 7.5], 10 mM magnesium acetate, 50 mM potassium glutamate, 0.1 mg/mL bovine serum albumin, and 2 mM ATP), heated for 3 min at 95˚C, and cooled in chilled water. A portion was analyzed using 9% polyacrylamide gel electrophoresis, followed by fluorescence imaging. In lanes 3 and 4, minor DNA species that migrated most slowly are presumable oligomers of the reaction intermediates.


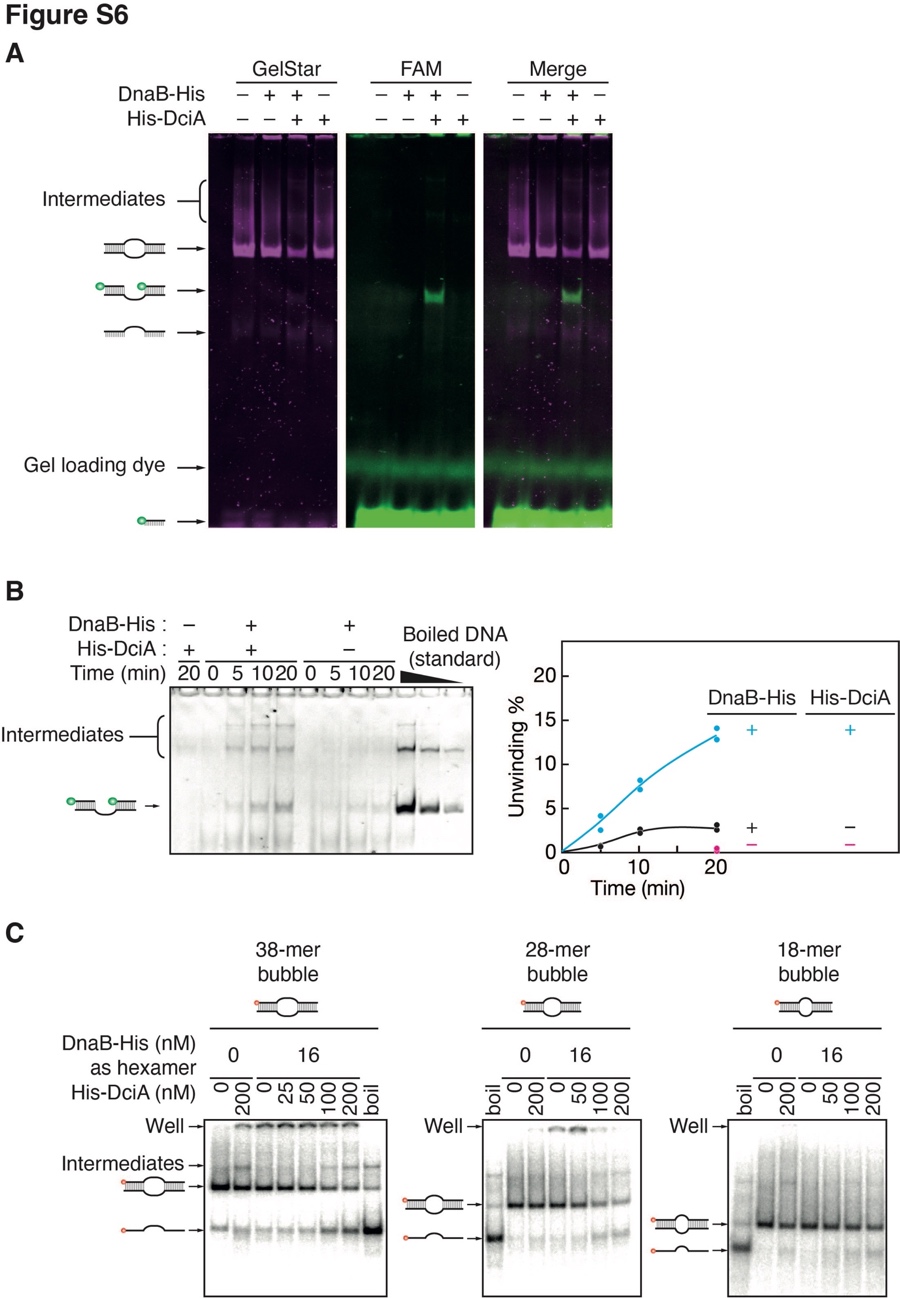


**Supplementary Figure S6. DNA helicase assays using a bubble DNA substrate**

(A-B) The DNA substrate with a 38-mer ssDNA bubble was incubated for 20 min (A) or the indicated time (B) with a FAM-labeled competitor ssDNA in the presence (+) or absence (–) of DnaB-His (0 or 16 nM as hexamer)/His-DciA (0 or 100 nM as monomer). The products were analyzed on 9% polyacrylamide gel. After the FAM image was taken, the gel was soaked in buffer containing GelStar Nucleic Acid Gel Stain (Lonza), followed by fluorescence imaging. False color images of FAM (magenta) and GelStar (green) as well as the merged image of the two are shown. The DNA structures are indicated schematically, where green circles indicate FAM labelled DNA ends.

(C). DnaB-His and His-DciA were incubated with the DNA substrate with a different size of ssDNA bubble (38-, 28-, or 18-mer). The products were analyzed on 9% polyacrylamide gel. The DNA structures are indicated schematically, where red circles indicate radioactively labelled DNA ends. The gel images were used to draw reaction curves for *Figure 6E*.

**
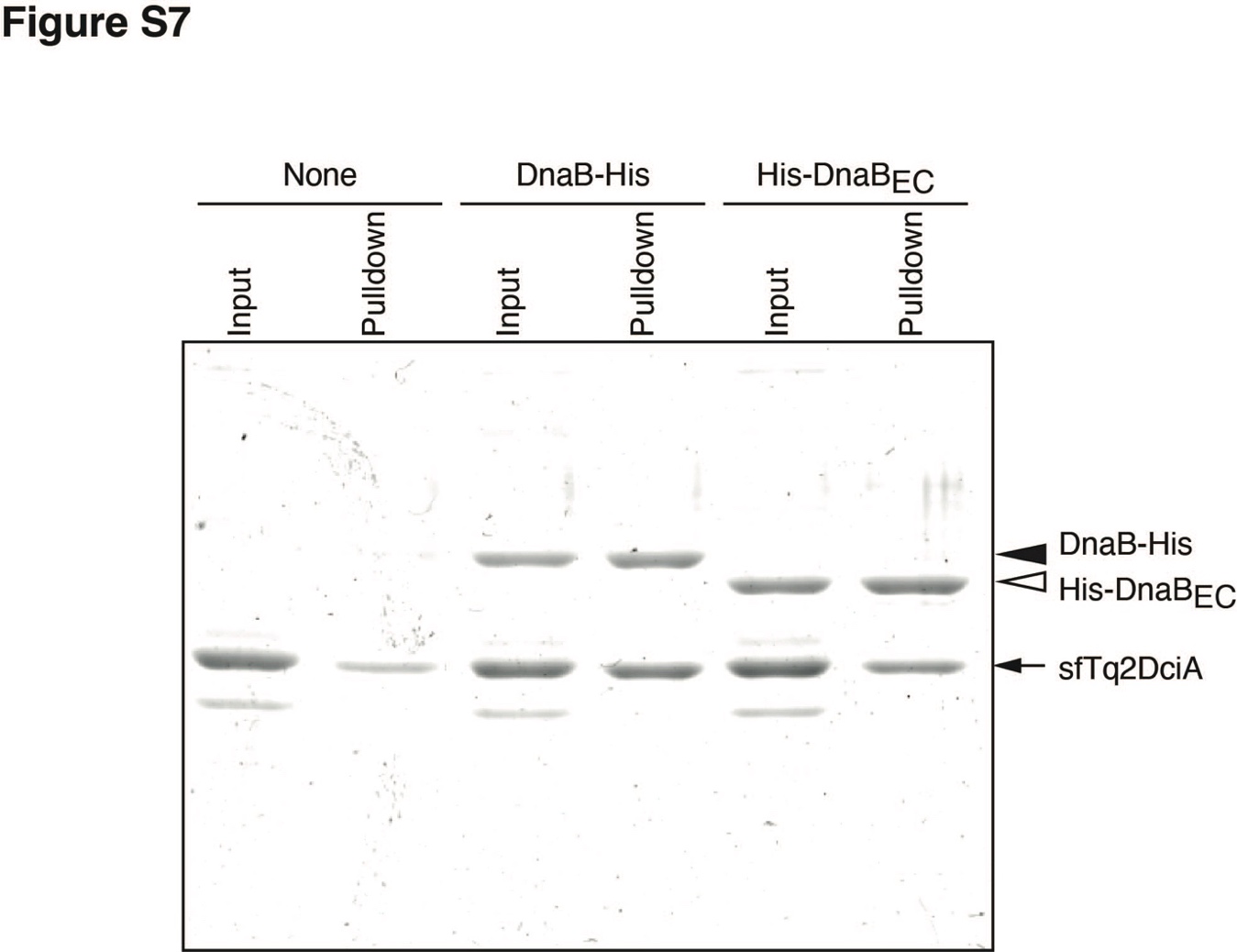
**

**Supplementary Figure S7. A pulldown assay using *E. coli* His-DnaB_EC_**

The interaction between purified sfTq2-DciA and *E. coli* His-DnaB_EC_ was assessed using a pulldown assay. When sfTq2-DciA and *E. coli* His-DnaB_EC_ or *Caulobacter* DnaB-His were incubated as described in the legend for *Figure 7B*, we observed co-elution of sfTq2-DciA with DnaB-His, but not with *E. coli* His-DnaB_EC_ (data not shown), suggesting that the affinity of sfTq2-DciA for *E. coli* His-DnaB_EC_ is weaker than that for His-DnaB. Because reaction and washing buffers contained 300 mM and 400 mM NaCl, respectively, we reasoned that these high salt concentrations are too stringent to detect the moderate interaction between sfTq2-DciA and His-DnaB_EC_. Therefore, we repeated the assay using R2 buffer containing 100 mM NaCl for the reaction and W2 buffer containing 300 mM NaCl for washing beads. A representative gel image from several independent experiments was shown. Under these ‘less stringent’ conditions, His-tagged protein-independent recovery of sfTq2-DciA was seen at a background level (lane 2). The recovery of sfTq2-DciA was greatly increased by co-incubation with DnaB-His (lane 4). Similarly, we observed a modest, but reproducible increase in sfTq2-DciA recovery together with His-DnaB_EC_ (lane 6). Taken together with the finding that His-DciA can stimulate helicase loading of *E. coli* DnaB (*Figure 6*), these results suggested that DciA operates *E. coli* DnaB through direct interaction.

Buffers used are as follows: R2 buffer (25 mM Tris HCl [pH 7.5], 10 mM magnesium acetate, 100 mM sodium chloride, 0.01 mM ATP and 5% glycerol). W2 buffer (25 mM Tris HCl [pH 7.5], 10 mM magnesium acetate, 300 mM sodium chloride, 0.01 mM ATP, 40 mM imidazole and 5% glycerol). *E. coli* His-DnaB_EC_ was purified previously (Hayashi et al., 2020. J. Biol Chem. DOI: 10.1074/jbc.RA120.014235).
